# Laboratory Efficacy of Locally Available Backwashing Methods at Removing Fouling in Hollow-Fiber Membrane Filters Used for Household Water Treatment

**DOI:** 10.3390/membranes11050375

**Published:** 2021-05-20

**Authors:** Camille Heylen, Alice Oliveira Aguiar, Gabrielle String, Marta Domini, Nathaniel Goff, Anna Murray, Ayse Asatekin, Daniele Lantagne

**Affiliations:** 1 Department of Civil and Environmental Engineering, Tufts University, Medford, MA 02155, USA; gabrielle.string@tufts.edu (G.S.); marta.domini@unibs.it (M.D.); zutana@gmail.com (A.M.); daniele.lantagne@tufts.edu (D.L.); 2 Department of Chemical and Biological Engineering, Tufts University, Medford, MA 02155, USA; aliceoa00@gmail.com (A.O.A.); nathaniel.goff@tufts.edu (N.G.); ayse.asatekin@tufts.edu (A.A.)

**Keywords:** backwashing, fouling, household water treatment, humanitarian response, membrane filters

## Abstract

Hollow-fiber membrane filters (HFMFs) for household water treatment (HWT) can efficaciously remove disease-causing organisms in laboratory settings. However, lower effectiveness in use in low- and middle-income countries (LMICs) and humanitarian contexts (HCs) has been observed and attributed to membrane fouling and the associated cleaning. In LMICs/HCs, it is not possible to prevent and control fouling using commonly known methods (e.g., testing influent water, maintenance regimes), and the literature on fouling/cleaning of HFMFs distributed in LMICs is scarce. As such, controlled laboratory experiments were conducted to determine the efficacy of locally available (in LMICs/HCs) backwashing solutions at removing fouling using different influent waters and HFMF types. Four commonly distributed HFMFs were selected; fouling layers were developed by filtering three influent water compositions, representing LMIC/HC waters, for 10-days, and bleach, water, or vinegar backwashing solutions were used for daily backwashing. Filter performance indicators included: fiber mechanical properties (strain at break, break force), water quantity performance (flow), water quality performance (turbidity, *E. coli*), and imaging. The study found fouling developed rapidly and altered mechanical properties and water quantity indicators within 200 h of filtration. Fouling did not decrease water quality indicators. Backwashing improved the filter’s mechanical properties and water quantity performance, but it did not fully recover the initial performance. Additionally, recovery differed between backwashing solutions, and no universal cleaning recommendation appropriate for HFMFs in LMICs/HCs was identified. Overall, fouling development and control depended on HFMF type, influent water quality, and backwashing solution type; thus, caution before distributing HFMFs for long-term use in LMICs/HCs is recommended.

## 1. Introduction

In 2017, an estimated 29% of the global population did not have access to safely managed water supplies, and 785 million people lacked even a basic drinking service [1]. While one aim of the United Nations Sustainable Development Goals is to ensure universal and equitable access to safely managed and affordable drinking water for all [1], household water treatment (HWT) options are currently promoted as an interim solution for those without safe drinking water [2]. 

HWT methods, such as boiling, chlorination, flocculant/disinfectant powder, solar disinfection, and filtration, have been shown to improve microbiological water quality and reduce diarrheal disease among users [3,4]. Among filtration methods, there has been extensive previous research and promotion of sand and ceramic filters [5,6]. More recently, hollow-fiber membrane filters (HFMFs) have been increasingly promoted. HFMFs consist of several hundred tubular fibers, with specified pore sizes, packed together. Water flows into the filter casing, through the porous membrane walls into the cores, and into a storage container. With advertised pore sizes between 0.1 and 0.02 μm [7], HFMFs remove >99% bacteria from contaminated water in laboratory settings [8]. As such, HFMFs can obtain at least a targeted protection rating from the WHO Certification Scheme for household treatment products without any other treatment [8]. 

In Rwanda, one HFMF was shown to reduce diarrhea in users by 78% 12–18 months after distribution [9]; in the Democratic Republic of the Congo, HFMF use reduced diarrhea by 15% [10]. However, microbiological water quality improvement was lower than anticipated in both studies, as 29% and 36% of intervention households had detectable thermotolerant coliforms (TTCs) in filtrated water. HFMF effectiveness in removing microbiological contaminants was also lower than laboratory efficacy in other field studies. In Kenya, between 18 and 26 months after filter distribution, a geometric mean of 20.6 *E. coli* CFU/100 mL was observed in filtered water [11]. In Honduras, 52% of filters tested after 23 months of use had filtrate with >10 *E. coli* CFU/100 mL [12]. In South Sudan, 38–40% of filtrate samples were ≥1 TTC CFU/100 mL after the second visit for filters cleaned after previously having contaminated filtrate [13]. In Fiji, 71% of filtered water samples had microbiological contamination [14]. Other reported concerns included misuse, broken/missing parts without access to replacements, slow flow, difficulty with backwashing (due to lost parts, difficulty in use, or need for clean water), and bad water smell/taste [11,14,15,16]. Thus, there appears a consistent disparity between laboratory efficacy and field effectiveness results when using HFMFs for HWT in low- and middle-income countries (LMICs) and humanitarian contexts. 

One potential reason for the discrepancy in microbiological membrane performance between laboratory and field results is differences in membrane fouling and the associated mechanisms to control fouling [13,16]. Fouling is the adhesion and accumulation of influent water components on membrane surfaces that can cause a decline in effluent quality and quantity [17,18,19] and changes in membrane material properties [20,21]. Several principal fouling mechanisms have been identified, including [17,22] physical fouling, such as pore blockage by colloids of similar size to the pores, or cake formation, formed by larger particles depositing on the membrane surface; chemical fouling, such as organic adsorption (e.g., humic or fulvic acids), and inorganic precipitation (due to pH change, hydrolysis, or oxidation of Ca, Mg, CO_3_, SO_4_, Si, and Fe compounds); and biological fouling caused by microorganisms that adhere to the membrane surface and grow to create a biofilm. Fouling can be reversible, where particles are removed by physical processes, or irreversible, where the material is adsorbed to the pores and requires chemical cleaning to recover performance [23]. Fouling is influenced by water chemistry, membrane properties, temperature, mode of operation, and hydrodynamic conditions [17,22,23]. While membrane fouling has been extensively investigated in other applications, and it is known that membranes foul over time [24], questions remain on how quickly fouling occurs on HFMFs for HWT in LMICs and humanitarian contexts and its reversibility by using locally available backwashing solutions. Membrane fouling can be controlled via design and operation, pretreatment of influent water, and regular maintenance (e.g., daily backwashing/forward flushing, chemical cleaning with alkalis or acids [23,25]). HFMFs use membranes initially developed for use within well-controlled, large-scale water treatment facilities operated by experts to manage influent water quality. A small-scale application of HFMFs is within cartridges designed for short-term use (e.g., treating surface water while camping). These small-scale cartridges have also been widely distributed for free to families living in LMICs and humanitarian contexts for HWT, intended to last a family for many months to years. In these contexts, it is not feasible to optimize a site-specific filter design, pretreat water, perform intensive maintenance using clean solutions and without potentially damaging fibers, or know when an HFMF has failed. To control fouling, some HWT HFMF manufacturers recommend forcefully backwashing or soaking the filters with clean water, diluted chlorinated solution, vinegar, salt water, or hot water [26,27,28]. Manufacturers claim that HFMFs can be “cleaned and reused almost indefinitely” [29] and used for “an extended period of time (years) if properly maintained” [30]. However, laboratory evidence to support using these recommended locally available cleaning mechanisms to recover membrane performance while maintaining filter function is lacking.

With the goal of providing recommendations for the backwashing of HFMFs to inform HFMF distribution programs and membrane applications in LMICs and humanitarian contexts, this research explores how fouling caused by different influent water qualities and backwashing with locally available solutions impact the filters’ mechanical properties, water quantity, and quality performance. 

## 2. Materials and Methods

To complete the research, (1) four commonly-distributed HFMFs were selected and purchased; (2) smaller modules were assembled for testing; (3) a system was built to test HFMFs with three influent water types; (4) a system was built to simulate backwashing with three cleaning solutions; (5) three data collection runs were conducted, including fouling (filtering with influent waters), influent and effluent water quality testing, and fouling removal (backwashing); (6) postmortem SEM and tensile strength tests were conducted; and (7) data were analyzed. Each step is described below. In total, four HFMF filters, three influent waters, and three backwashing solutions (plus no-backwashing control) were tested in duplicate for a total of 96 modules.

### 2.1. Selection of Commonly Distributed HFMFs

Four different commonly distributed HFMFs that met the minimum criteria for HWT filters were purchased [7]. All HFMFs consisted of fibers bundled in a U-shape inside a plastic casing, and water moved via gravity flow from outside to inside the fiber during treatment. Filters 1, 2, and 3 (F1–3) consisted of plastic-encased 0.1-μm pore size microfiltration (MF) hollow fibers, with microporous outer surfaces and relatively symmetric cross-sections. For F1–F2, no pretreatment mechanism was provided. A 75-micron foam prefilter was included with F3. A 50–60 mL syringe to complete backwashing and a water storage container were provided with all three filters. Filter 4 (F4) consisted of 0.02-μm ultrafiltration (UF) membranes with a highly asymmetric cross-section and a dense outer selective layer encased in a plastic structure. The structure also contains an 80-μm prefilter, a pump for backwashing filtered water, and a water storage area.

### 2.2. Preparation of Modules for Testing

To prepare the test modules, plastic filter casings were carefully opened using a circular saw. Individual fibers were gently detached at the base with a razor. To obtain 24 modules of each HFMF type with similar flow rates, 3 (F1 and F3), 4 (F2), and 15 (F4) fibers were potted in a U-shape (F1–3) or straight (F4) into 1/4′′ internal diameter (ID) semiclear crack-resistant polyethylene tubing and glued to create a seal without blocking fiber pores (Figure S1). The average fiber lengths for F1–4 were 33, 38, 41, and 186 cm, respectively. Modules were then tested to verify their integrity and ensure similar flow by filtering deionized (DI) water for one hour at 1 psi transmembrane pressure and weighing the effluent. Modules with the most similar initial flow (Q_0_ determined with Equation (S2); SI) were selected for inclusion. 

### 2.3. Filtration System 

Four filtration units, each holding eight modules from the same HFMF, were built to ensure a continuous flow of influent water through modules (Figure S2a). Each system consisted of a plastic 20-L tank connected to the modules through a network of 1/4′′ ID pipes and manifolds, regulated by an air regulator (0.5–3.5 psi outlet pressure range) and a pressure gauge (Kodiak Controls KC25-3# Low-Pressure Gauge 3 psi) to maintain a constant transmembrane pressure of 0.5–1 psi, without air pockets. This pressure approximated a filled 5-gal bucket head pressure with a 1 ft tube, a common implementation of HFMFs in households. Tanks were placed on a stir plate to avoid the settling of influent water particles and bacteria. Before an experimental run, the system was cleaned with 5% sodium hypochlorite, rinsed with Milli-Q^®^ water to remove any chlorine, and left to air dry. Complete rinsing of chlorine from the system was confirmed (free chlorine residual equal to 0 mg/L) using a Lamotte colorimeter (Lamotte, Chestertown, MD, USA).

Fouling experiments were run with three influent water compositions. Influent #1, simulating biological fouling and termed “Bacteria”, consisted of Milli-Q water buffered with phosphate-buffered saline (PBS) solution with 10^6^ CFU/100mL *E. coli* (ATCC 11220). *E. coli* cultures were prepared from streak plates in Luria−Bertani broth using standard methods [31]. *E. coli* cell concentrations in exponential phase cultures were estimated through turbidometry using a spectrophotometer (OD = 600 nm; GeneQuant100, GE Healthcare Life Sciences, Hatfield, UK) as per Standard Methods [31] and used to calculate the spike culture volume into 20 L buffer solution. Influent #2 simulated combined biological and chemical fouling and was termed “BacChem”. It consisted of Influent #1 with the addition of 0.63 g/20 L humic acid (HA; 6813-04-4, Alfa Aesar, Haverhill, MA, USA) and 1.1 g/20 L CaCl_2_ (C20010–1000.0, Research Products International Corporation, Madison, WI, USA) to obtain theoretical total organic carbon (TOC) and Ca^++^ concentrations of 15 mg/L. Influent #3 simulated combined biological and physical fouling and was termed “BacSed”. It was prepared by adding 2.2 g/20 L sediment (ISO spec. 12103-A2 fine test dust) to Influent #1 to reach 30 NTU turbidity. Fine test dust includes particles larger and smaller than HFMF pores (0.97–124.50 μm) and is composed of 77–91% silica and aluminum and traces of Fe, Na, Ca, Mg, Ti, and K. 

### 2.4. Backwashing System 

The backwash system consisted of three 1-L tanks filled with cleaning solution, connected to four modules via 1/4′′ ID pipes, and an air regulator (0.5–10 psi outlet pressure range) and a pressure gauge (Kodiak Controls KC25-3# Low-Pressure Gauge 10 psi) to maintain a constant backwash pressure of 5 psi (Figure S2c).

Three locally available cleaning solutions in LMICs and humanitarian contexts were tested: bleach, vinegar, and clean water. Bleach solution was prepared by diluting 6% laboratory-grade commercial bleach (Pure Bright, KIK International, Houston, TX, USA) to 0.5% sodium hypochlorite (NaOCl) with Milli-Q^®^ water. Concentration was confirmed using iodometric titration (Hach Method 8209, Loveland, CO, USA). This 0.5% concentration was selected because a 10:1 dilution is simple to prepare, a 0.5% solution efficaciously removes *E. coli* from surfaces [32], and it was between the HFMFs’ manufacturer-provided ranges [26,27]. Commercial vinegar (6% acetic acid; Heinz, Pittsburgh, PA, USA) was purchased and used without modification, as recommended by manufacturers [27]; it is available in LMICs and humanitarian contexts. DI water was used without modification as clean water.

### 2.5. Data Collection Runs

Each module was exposed to daily fresh influent water for 17.5 h/day for 10 consecutive days and backwashed for 5 min/day. This aimed to simulate a timeframe of ~3 months of normal use (2.5 h/day) with cleaning once per week, a timeframe shown to be before membrane failure begins in South Sudan [13]. 

To ensure the consistency of the influent water, turbidity, *E. coli* concentration, total dissolved solids (TDS), and pH were tested at the beginning of daily filtration, and turbidity and *E. coli* concentration were tested again after filtration. Backwashing solution pH was also tested daily to ensure consistency.

Turbidity was measured with a calibrated Lamotte 2020we turbidimeter (Lamotte, Chestertown, MD, USA). Triplicate readings were averaged, with results of zero replaced with the minimum detection limit (0.05 NTU). *E. coli* concentrations were measured by preparing appropriate dilutions of the samples by filtering through a 0.45 µm membrane, plating on m-ColiBlue24^®^ (Hach, Loveland, CO, USA) media, and incubating at 35 °C for 24 h [31]. *E. coli* colonies were then enumerated and recorded. Plates with no detectable colonies were replaced with the minimum detection limit of 1 colony as a conservative estimate. The geometric mean of plates in the countable range (1–200 colonies) was calculated and reported in CFU/100 mL. TDS was measured with a PC60 Premium Multiparameter probe (APERA Instruments, Columbus, OH, USA). pH was measured with an Orion^®^ 9106BNWP probe (Thermo Electron Corporation, Waltham, MA, USA). 

During the first 14 h of filtration, effluent water was collected in nonsterilized 2-L HDPE collection bottles and weighed (Figure S2a). The pressure and exact duration of filtration were recorded. Then, effluent water was collected into sterile 118 mL WhirlPak™ bags (Nasco Company, Fort Atkinson, WI, USA) for *E. coli* and turbidity testing and an additional flow reading using the methods described above (Figure S2b). This “second” filtration lasted ~3.5 h, depending on flow rates, as a minimum volume of 118 mL was needed to conduct reliable *E. coli* testing. Each module was tested for *E. coli* and turbidity every other day, and when the effluent collected the day before showed *E. coli* contamination breakthrough. 

After filtration each day, all noncontrol modules were backwashed using the backwashing system and their assigned backwashing solution. Between backwashing and restarting filtration, modules were filled with PBS solution to maintain a wetted state to avoid damaging fibers and prevent the bacteria from desiccating. Nonbackwashed control modules were similarly maintained in PBS between filtration cycles.

**Figure 1:**
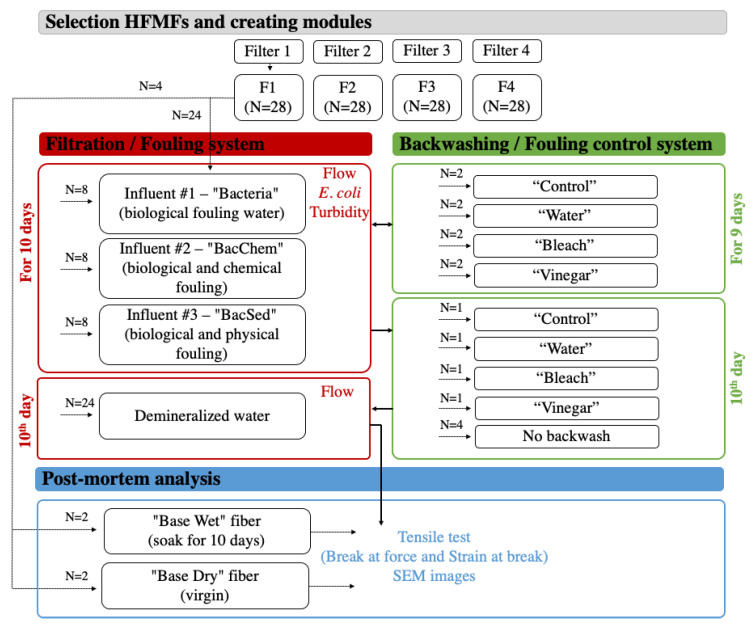
Experimental design. The filtration/backwash cycle was repeated for nine consecutive days. On the tenth day, filtration was performed, but only one module of each pair was backwashed after filtration. The other module from each pair was used as no-backwash control to assess the impact of the final study-end backwash. Finally, all modules were run with demineralized water and proceeded to postmortem analysis.

The filtration/backwash cycle was repeated for nine consecutive days (Figure 1). On the 10th day, only one duplicate was backwashed after filtration, and a final filtration with DI water was performed to prepare the modules for postmortem analysis. 

### 2.6. Postmortem Analysis

After each run, modules were carefully cut open and the fibers separated for postmortem analyses. The fibers were left to dry at ambient temperature overnight (for F1–3) or for 24 h (F4) due to the larger fiber volume (Figure S3). After drying, all fibers were stored at 4 °C. To assess the mechanical properties, tensile tests were conducted using an ARES-LN2 rheometer (TA Instruments, New Castle, DE, USA) on at least three fibers per module at room temperature. Fibers were clamped at both ends and pulled at a constant elongation velocity of 0.02 mm/sec until failure; break force was measured, and strain at break (Equation (S1) in SI) was then calculated. These parameters relate to membrane material properties, and a decrease of these coupling parameters could indicate an increase in toughness and fiber embrittlement [21]. Tests were also conducted on two additional modules of each membrane type soaked in DI water (no filtration) over the 10-day experimental period, to be used as a baseline (“Base Wet”) in postmortem analyses, as well as on unsoaked fibers (“Base Dry”) (Figure 1). Please note that burst pressure was not assessed because the membranes are porous and somewhat anisotropic; as such, break force is a reasonable proxy to burst pressure. 

Fiber morphology was characterized by scanning electron microscopy (SEM) using a Phenom G2 Pure SEM (Phenom-World BV, Eindhoven, The Netherlands) operating at 5 kV. Fiber cross-sections were obtained by freeze-fracturing in liquid nitrogen. Before imaging, all samples were coated with gold-palladium for 90 s using a Cressington Sputter Coater 108 (Cressington Scientific Instruments, Watford, UK).

### 2.7. Data Analysis

Data were entered into Microsoft Excel (Redmond, WA, USA) and analyzed with R (RStudio, Vienna, Austria). Qualitative SEM analysis was used to confirm fouling at the study’s end by imaging control (nonbackwashed) modules and backwashed modules, with and without a final backwash. Indicators developed to assess impacts of fouling and backwashing included (equations in SI): strain at break and break force (mechanical properties), normalized flow (water quantity performance), and *E. coli* log reduction value (LRV) and turbidity reduction value (TRV) (water quality performance). Baseline and endline were defined for each indicator and used to calculate the relative change in performance over the length of the study (Table 1; Equation (S5)).

First, to determine if MF filters could be combined for analysis, a Kruskal–Wallis test was used to assess differences in select baseline and all endline indicators between: all filters (F1–F4); MF (F1–F3) filters only; and averaged MF filters (F1–3avg) and F4 (UF). Based on the results, MF filter results were combined and averaged for subsequent analysis. Then, a Kruskal–Wallis test was used to compare “Base Dry” to “Base Wet” break force and strain at break for each individual filter to assess the impact of wetting.

To confirm fouling on nonbackwashed modules, a Wilcoxon signed-rank test was used to compare baseline to endline indicators for MF (F1–3avg) and UF (F4) filters. To assess fouling consequences on nonbackwashed modules, a Kruskal–Wallis test was used to compare the endline indicators between influent water conditions (Bacteria, BacChem, and BacSed) for MF (F1–3avg) and UF (F4) filters. 

To assess the backwashing impact, a Kruskal–Wallis test was used to compare endline indicators between backwashed and nonbackwashed filters for each influent water condition (Bacteria, BacChem, and BacSed) stratified by filter type (MF (F1–3avg) and UF (F4)). Lastly, to assess the impact of solutions on backwashed filters, a Kruskal–Wallis test was used to compare the endline indicators between solution types for each influent water condition (Bacteria, BacChem, and BacSed), stratified by filter type (MF (F1–3avg) and UF (F4)). For all statistical tests, *p* < 0.05 was significant.

## 3. Results

Across the three influent waters and eight modules tested for each of four HFMFs (96 modules total) for 10 days, the testing parameters obtained in the actual experiments were as planned in the Methods section except for time of filtration and applied pressure (Table S1). The time of filtration increased in the BacChem and BacSed influent waters due to fouling, and applied pressure was lower in F4 than F1–3 because a larger number of fibers were not in a U-shape in the module. Additionally, 4 of the 96 modules were removed from the analysis because of leakage (two F4/BacChem/Water, one F4/Bacteria/Bleach, and one F2/BacSed/Control). Thus, results from 92 modules are presented below. 

### 3.1. Assessing Filter Variation

While the indicators tested (strain at break, break force, normalized flow, *E. coli* LRV, TRV) varied between the four HFMFs (F1–F4), the variation was smaller between F1–F3 than between F1–3_avg_ and F4 (Table S2). The average inner and outer diameters of Base Dry fibers before testing were measured by SEM to be 237 ± 16 and 406 ± 16 μm, respectively, for F1–3_avg_ and 584 ± 13 and 926 ± 16 μm for F4. Before testing, both strain at break and initial break force for Base Dry fibers were higher in F4 than F1–3_avg_ (27–29% for F1–3_avg_ and 51% for F4, *p* = 0.003; 300–331 mN for F1–3_avg_ and 1610 mN for F4, *p* = 0.004) (Table S2). Over 10 days of filtration, across all influent waters and backwashing methods, significant differences between F1–3_avg_ and F4 were observed for normalized flow (0.16–0.20 for F1–3_avg_ and 0.67 for F4, *p* < 0.001), *E. coli* LRV (5.3–5.4 for F1–3_avg_ and 5.6 for F4, *p* < 0.001), strain at break (13–15% for F1–3_avg_ and 24% for F4, *p* < 0.001), and break force (274–289 mN for F1–3_avg_ and 1716 mN for F4, *p* < 0.001) but not between F1, F2, and F3 for normalized flow (*p* = 0.14), *E. coli* LRV (*p* = 0.71), and strain at break (*p* = 0.43). The difference between F1–3_avg_ and F4 was not unexpected as F1–F3 were all microfiltration hollow-fibers (MF membranes), and F4 was made of ultrafiltration hollow-fibers (UF membranes). MF and UF membranes have different properties (including pore size, thickness, mechanical strength, and surface properties) that influence the filters’ performance, such as flow rate and removal efficacy [33]. Although the obtained results varied slightly between F1–F3, the values were sufficiently similar to be combined as an average for subsequent analysis and presentation here. These are heretofore referred to as MF (F1–3_avg_) and UF (F4).

### 3.2. Influence of Fiber Wetting Before Testing

The strain at break for Base Dry fibers was, on average, between 27–29% for F1–3_avg_ and 51% for F4, and the average break force ranged between 300–331 mN for F1–3_avg_ and 1610 mN for F4 (Table S3; Figures S7 and S8). For Base Wet fibers, significant increases were observed in the strain at break for F2 and F3 (*p*-values = 0.016, 0.028) and the break force for F4 (*p* = 0.004). These results suggest improvements in mechanical properties due to fiber wetting, but more investigation is needed to confirm this observation and to describe the impact of wetting and the potential for continuous wetting in LMICs and humanitarian contexts. Please note that water quantity and quality indicators were not tested with Base Dry and Wet modules.

### 3.3. Development of Fouling on Membranes without Backwashing

**Figure 2:**
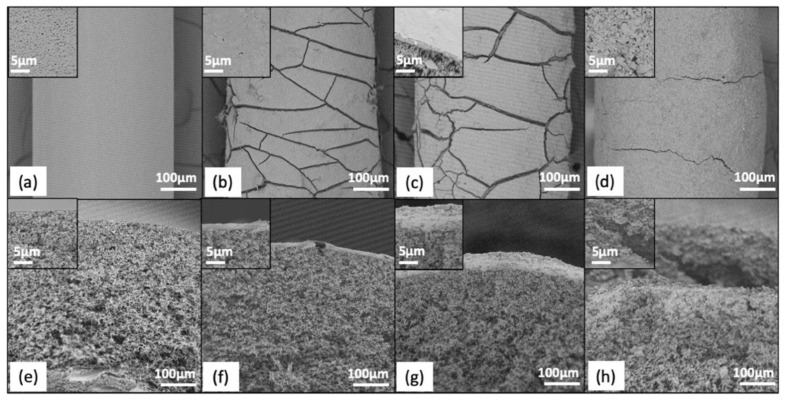
SEM images of fouling development on F2 for control modules (nonbackwashed) by influent water. Outer membrane surface at 500× and 15,000× (insets) for control: (**a**) clean fiber, (**b**) Bacteria, (**c**) BacChem, and (**d**) BacSed. Cross-section showing fouling layer thickness on the outer surface (2500× and 10,000× (insets)) for (**e**) clean fiber, (**f**) Bacteria, (**g**) BacChem, and (**h**) BacSed.

Fouling was confirmed by comparing the baseline indicators to the endline indicators for control modules (modules that were not backwashed but had influent waters filtered through them for 10-days) (Table S4). SEM images also supported the presence of fouling (Figure 2 and Figure S9). Significant differences were seen at the endline for strain at break, break force, and flow for MF and UF membranes (six *p*-values, *p* < 0.017 across indicators) but not for *E. coli* LRV or TRV (four *p*-values, *p* > 0.195 across indicators) (Table S4).

**Figure 3:**
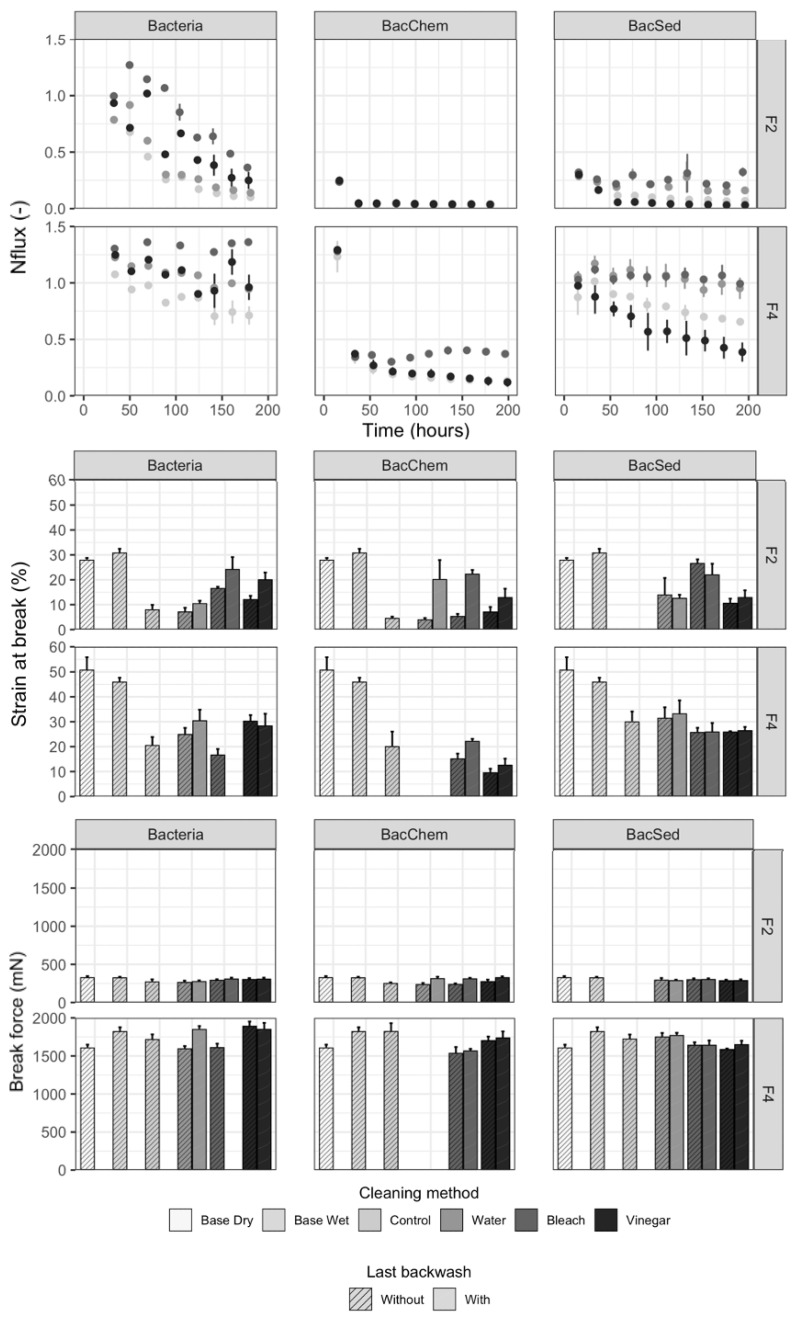
Normalized flow, strain at break, and break force for MF membranes (F2) and UF membranes (F4) by influent water (Bacteria, BacChem, and BacSed), backwashing solutions (colors: controls, water, bleach, and vinegar), and backwashing completion (shade-in, without and with a last backwash). Results for F1 and F3 are similar and presented in the supporting information.

As fouling developed, mechanical property measures decreased (Figure 3, Figures S7 and S8). On average, both strain at break and break force decreased at endline for nonbackwashed modules compared to Base Wet fibers (strain at break: 76% decrease (MF), 48% (UF); break force: 16% (MF), 4% (UF)) (Table S4; Figure 5a). Strain at break was significantly different between influent water conditions (2 *p*-values, *p* < 0.001) (Table S5; Figure S7), and break force was significantly different between influent water conditions for MF (*p* < 0.001) but not for UF (*p* = 0.082) (Table S5; Figure S8).

Water quantity performance also declined with fouling (Figure 3 and Figure S4). Flow significantly decreased between baseline and endline for nonbackwashed modules by, on average, 93% (MF) and 47% (UF) (2 *p*-values, *p* < 0.001) (Table S4; Figure 5a). Flow reduction was statistically different between influent waters for each filter type (2 *p*-values, *p* < 0.001), with lower average flow values for the BacChem influent water condition (Table S5; Figure S4). 

SEM images also showed differences in fouling layers between the influent waters. The BacChem influent layer was thicker than that of the Bacteria influent (Figure 2 and Figure S9). The BacSed influent led to the thickest fouling layer, but this layer appeared to be somewhat porous. 

Conversely, fouling did not, over the 10-day study period, impact *E. coli* removal: all membranes removed an average of 5.0–5.9 LRV *E. coli* irrespective of influent water or HFMF type (Tables S4 and S5; Figure S5). Turbidity reductions were greatest with higher initial influent water turbidity but did not change over the course of the 10-day study (Tables S4 and S5; Figure S6).

These results confirmed the presence of fouling after 10 days (~200 h) of filtration. Flow reductions were consistently observed, as well as strain at break reductions, indicating that fibers became more brittle. This result is consistent with other studies that attribute this to wear-and-tear during the filtration process [21,34]. Additionally, membrane fouling mechanisms depended on HFMF type, and more adverse outcomes for MF membranes were observed, which suggests different fouling mechanisms between MF and UF due to differences in fiber material and properties. Influent water characteristics also influenced membrane-fouling mechanisms, as predicted and observed in other membrane applications [17,25]. Visible differences in fouling layers between the influent waters help further understand the quantity performance decrease over time. The filtration of BacChem influent water causes the formation of a thick and dense fouling layer, which consequently led to rapid decreases in flow. The filtration of the BacSed influent led to the thickest yet visibly more porous fouling layer (perhaps because larger particle sizes aggregated together) than the BacChem layer, so the flow decrease was less. Lastly, the Bacteria influent led to a thinner fouling layer; as such, flow decreases developed more slowly than in other water conditions. The thick and dense fouling layer in BacChem is attributed to the presence of humic acid; humic acid has been identified as a major contributor to irreversible fouling in both UF and MF membranes because it adsorbs to the membrane surface [23,35]. This effect was exacerbated in the presence of multivalent cations such as Ca^2+^, which reduce humic acid solubility and promote aggregation [17,36,37]. Conversely, fouling did not impact filter quality performance. Previous studies have found that fouling can improve *E. coli* retention by reducing the pore size on membrane surfaces [38] or reduce *E. coli* retention when changes in mechanical properties cause fiber breakage and breakthrough. Breakthrough was not observed in our study, which could be due to the short duration. A summary of fouling outcomes, presented as the relative change from baseline to endline, is provided in Figure 5a.

### 3.4. Performance of Backwashed Modules Compared to Nonbackwashed Modules

Impacts of fouling removal from backwashing was assessed by comparing backwashed modules to control modules with the same indicators as above: mechanical properties (strain at break and break force; discussed separately for MF and UF), water quantity performance (normalized flow), and water quality performance (*E. coli* LRV and TRV).

In terms of fiber mechanical properties, backwashing significantly increased strain at break for MF compared to the control nonbackwashed modules (three *p*-values, *p* < 0.001 stratified by influent water conditions) (Table S6; Figure 3 and Figure S7). Average strain at break was highest for backwashed modules for Bacteria and BacSed than BacChem influent waters (Table S6). However, backwashing MF for 10 consecutive days did not fully recover strain at break, which remained, on average, 40–52% lower for MF than Base Wet fibers across all influent waters (Figure 5a). Backwashing UF did not significantly change strain at break for backwashed modules as compared to control modules (3 *p*-values, *p* > 0.09, stratified by influent water conditions) (Table S6; Figure 3 and Figure S7). Therefore, strain at break was still 36% lower compared to “Base Wet” fibers (Figure 5a). Membrane strain at break also increased, on average, with the final study-end backwash (on the 10th day) as compared to membranes that were not backwashed at study-end, indicating that backwashing even improved strain at break as the fibers were more ductile (Table S8; Figure 3 and Figure S7). 

Break force results were somewhat similar to strain at break results. Backwashing increased the break force for MF across influent water conditions compared to the control modules (3 *p*-values, *p* < 0.001, by influent water conditions) (Table S6; Figure 3 and Figure S8). Break force was, on average, highest for Bacteria and BacSed than BacChem. However, backwashing did not significantly increase the break force for backwashed UF modules compared to controls (3 *p*-values, *p* > 0.051, by influent water conditions) (Table S6; Figure 3 and Figure S8). Break force was, on average, 5–10% lower compared to the “Base Wet” break force for MF and UF after backwashing the fibers for 10 consecutive days (Figure 5a). Lastly, an increase of average break force in membranes with the final study-end backwash (on the 10th day) was not observed compared to membranes that were not backwashed at study-end (Table S8, Figure 3 and Figure S8).

Backwashing increased water quantity performance for all HFMFs across all influent water conditions as flow significantly increased for all backwashed modules compared to control nonbackwashed modules (6 *p*-values: *p* < 0.027 by filter type and influent water conditions, except for UF with BacSed, *p* = 0.164) (Table S6; Figure 3 and Figure S4). Flow was, on average, highest for Bacteria influent water conditions, then BacSed and BacChem for the backwashed modules (Table S6). However, backwashing did not fully recover the initial flow, and flow at the endline for backwashed modules was, on average, 75–82% lower for MF and 47% lower for UF than baseline (Figure 5a). Flow was not measured after the final study-end backwash (on the 10th day); thus, the impact of the final backwash was not assessed. 

Across all HFMFs and influent waters, backwashing did not affect water quality performance. Backwashing did not change *E. coli* removal for backwashed modules compared to nonbackwashed modules (6 *p*-values: *p* > 0.32 by filter type and influent water conditions except for MF and BacSed, where *p* = 0.003) (Table S6; Figure 5a and Figure S5). Additionally, backwashing did not affect TRV in the effluent for all backwashed modules compared to control modules (6 *p*-values: *p* > 0.44 by filter type and influent water condition, except for MF and BacSed, with *p* = 0.003) (Table S6; Figure 5a and Figure S6). Fiber breakage due to backwashing was not observed in our study, where a constant pressure of 5 psi was maintained during backwashing. 

Overall, backwashing (even once) improved filter performance indicators, particularly for MF. The lower effect of backwashing on the UF membrane may be explained by the lower impact of fouling, as described above. However, backwashing did not fully recover mechanical properties (strain at break and break force) or the initial flow rates (especially with certain influent water conditions) (Figure 5a). The decrease in strain at break indicates the fibers have become more brittle. When filters are backwashed under variable conditions, there is a risk that more brittle fibers will burst at higher pressures, subsequently allowing water to pass through the module unfiltered. This is a possible explanation for *E. coli* breakthroughs seen in field settings [12,13,14] but was not observed here with controlled backwashing pressures. Lastly, backwashing had a lower effect on fiber performance indicators associated with BacChem influent water, which may be attributed to the strong aggregation of humic acid and Ca^++^ on membranes and because more adverse outcomes were observed with this influent water due to fouling. 

### 3.5. Recovery of Filter Performance by Backwashing Solutions

When the results described in the previous section are further stratified by backwashing solutions (bleach, vinegar, or water), significant differences in mechanical properties and water quantity performance were observed between backwashing solutions (18 *p*-values: *p* < 0.001 by indicator, filter type, and influent water conditions, except MF and BacChem, where *p* = 0.651, 0.78, and 0.37 for flow, strain at break, and break force, respectively) (Table S7). Differences in water quality performance (*E. coli* removal or turbidity reduction) between backwashing solutions were not observed for any filter or influent water condition (12 *p*-values: *p* < 0.28 by indicator, filter type, and influent water conditions, except *E. coli* LRV and BacSed, where *p* < 0.001 for MF and UF) (Table S7; Figures S5 and S6) and, thus, are not described further. Overall, no backwashing solutions recovered flow and strain at break at endline when compared to baseline (Figure 5b).

Using bleach (0.50 ± 0.03% of sodium hypochlorite, pH = 11 ± 0.9) for backwashing improved the flow more than vinegar or water for most HFMFs and influent water conditions (Table S7; Figure 3 and Figure S4). Endline flow was, on average, between 4–96% lower than initial flow when backwashing with bleach, 55–96% lower with vinegar, and 31–96% lower with water (Figure 5b). Bleach backwashing also improved strain at break for two MF membrane influent water conditions (Bacteria and BacSed) compared to vinegar or water (endline strain at break was 19–32% lower with bleach, 39–61% lower with vinegar, and 42–68% lower with water) and recovered the break force for one MF membrane influent water condition (BacSed) (Figure 3, Figures S7 and S8). Flow increases (higher than baseline flow) occurred for one UF membrane influent water condition (Bacteria) using bleach backwashing, which suggests that bleach affected the fibers, perhaps through damage or increasing hydrophilicity (Figure 5b). Additionally, UF membranes cleaned with bleach exhibited lower recovery in break force and strain at break across influent water conditions. Therefore, although bleach, with its strong oxidizing potential [20,22], led to higher flow recoveries for all HFMFs and did not damage MF membranes, it might adversely impact the studied UF membrane.

The effectiveness of vinegar (6% of acetic acid, pH = 2.34 ± 0.09) as a backwashing solution is dependent on influent water composition and membrane type. The vinegar solution somewhat improved strain at break, break force, and flow of MF and UF membranes fouled with Bacteria influent waters compared to backwashing with water (Table S7; Figure 3, Figures S4, S7 and S8). Endline flow was 1–57% lower than initial flow when backwashing with vinegar (2–82% with water), strain at break was 39–61% lower with vinegar (42–68% with water), and break force was 1–9% lower with vinegar (4–14% with water) (Figure 5b). For BacChem influent waters, the vinegar solution only improved break force in UF membranes (compared to bleach). However, with BacSed influent water, vinegar reduced strain at break, break force, and flow for all HFMFs (Figure 3, Figures S4, S7 and S8), with indicators worse than those for nonbackwashed modules (Table S5), thus exacerbating fouling impacts (Figure 5b). Therefore, while vinegar was the best backwashing solution to improve mechanical properties with Bacteria influent water and to limit fiber brittleness (perhaps because vinegar can solubilize foulants and disrupt biofilms [39]), it was not an appropriate backwashing solution with BacSed influent water, maybe because of unknown interactions between influent water particles and membrane material properties.

Lastly, backwashing with water (pH = 6.6 ± 0.3) led to lower flow recovery than with bleach or vinegar for most HFMFs and influent water conditions (Table S7; Figure 3, Figure 5b and Figure S4). However, backwashing UF membranes exposed to BacChem and BacSed influent with water was the most effective method to recover the mechanical properties of the fibers (Table S7; Figure 3, Figures S7 and S8). Endline strain at break was 30–52% lower than initial strain when backwashing with water (43–76% with bleach and vinegar), and endline break force was 1–3% lower (6–15% with bleach and vinegar) (Figure 5b). 

**Figure 4:**
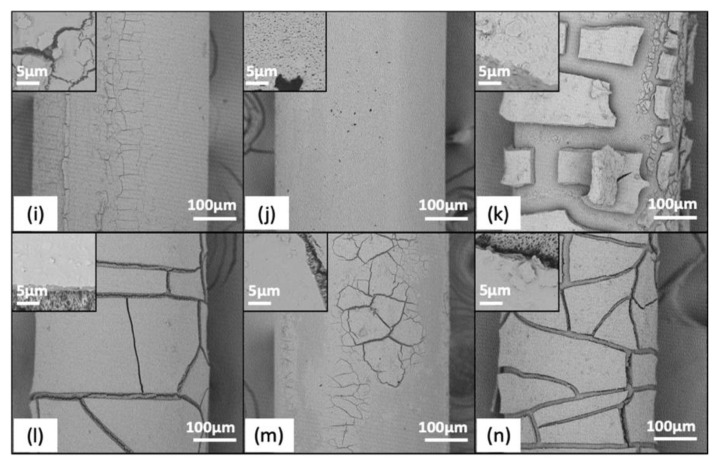
SEM images of fouling control on F2 for modules filtered with BacChem influent by backwashing solutions. Outer membrane surface at 500× and 15,000× (insets), showing fouling layer after BacChem filtration with final backwash using (**i**) DI water, (**j**) bleach, and (**k**) vinegar and without final backwash (**l**) DI water, (**m**) bleach, and (**n**) vinegar.

The effect of membrane backwashing with different backwashing solutions can also be further understood by evaluating the outer surface of the membranes through SEM at the end of the fouling cycle and then after a “last backwash” (Figure 4 and Figures S10–S13). For all influent waters, a fouling layer was clearly visible when the last backwash was not performed. While regular backwashing may have limited the growth of this layer, this was difficult to document given its variability along the length of each fiber and the limited sample size achievable by SEM. For MF membranes, the last backwash removed a visible fraction of the fouling layer on the membrane surface for Bacteria and BacChem influent waters, with bleach observed to remove the most fouling. Backwashing with vinegar and water for Bacteria and BacChem influent waters had variable observed efficacy but still worked better than no backwashing at all (controls; Figure 2b–d). Conversely, for the BacSed influent water, backwashing the membranes with bleach, water, and vinegar did not seem to noticeably impact the membrane fouling layer. All SEM images of these membranes showed a thick fouling layer, with no apparent difference between the membranes with and without the last backwash. Similar trends were observed for the UF membranes (F4), although differences were more difficult to visualize by SEM due to the smooth morphology of the membrane surface and the thinner fouling layers that formed.

## 4. Summary and Recommendations

**Figure 5:**
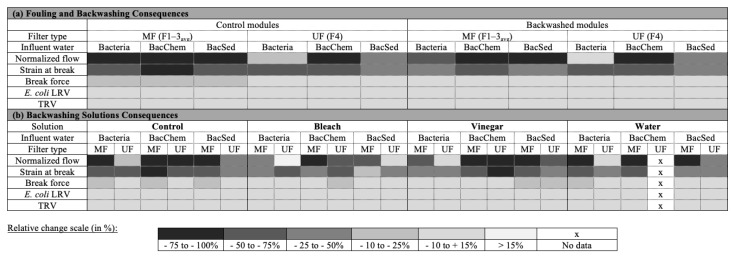
Relative change of indicators from baseline to endline (%, semiquantitative scale) for (**a**) control modules (fouling consequences) and backwashed modules (backwashing consequences) and (**b**) backwashed modules by backwashing solutions (backwashing solution consequences).

The effect of different influent water conditions and backwashing solutions was tested on fouling development and control for different HFMF types. Our results confirmed that fouling developed rapidly in HFMFs used with different influent waters and highlighted membrane fouling development and control depend on HFMF type, influent water characteristics, completion of backwashing, and backwashing solution type. A complete summary of fouling and cleaning consequences by assessed indicators, membrane types, influent water conditions, and cleaning solutions is provided in Figure 5. 

In this study, fouling rapidly decreased water quantity performance and mechanical properties (particularly, strain at break) but did not decrease water quality performance or cause fiber breakage. The lack of change in *E. coli* LRV and turbidity removal were attributed to not having fiber failures during the short study, regular cleaning with low backwash pressure, and/or unknown factors. Additionally, while all influent water led to flow reductions and changes in strain at break and break force across all filters, MF membranes were generally more impacted by fouling than UF membranes, and BacChem influent water (including bacteria, humic acid, and calcium) generally had more fouling consequences. 

For fouling control, the results indicate the importance of backwashing to recover water quantity performance and mechanical properties, especially for MF membranes. Backwashing depends on frequency and pressure (mainly removing surface deposits rather than internal foulants [22,40]), and, in this study, backwashing once every 17.5 h, with 5 psi pressure for 5 min, was insufficient to fully recover water quantity performance and the fiber’s mechanical properties. Optimizing these parameters may lead to better results, although the severity of fouling also depends on the concentration and nature of the membrane; complex regimes may not be possible for users to implement. Importantly, for the development of field-based cleaning recommendations, cleaning efficacy with the manufacturers’ recommended solutions (bleach, vinegar, and water) depended on influent water quality and membrane type, and there was no universal cleaning method that was appropriate for all fibers and influent water qualities. Water quantity performance recovery was generally better with bleach than other cleaning methods for MF membranes for all influent waters, but 0.5% sodium hypochlorite bleach possibly degraded the UF membrane’s mechanical properties. For all HFMFs, 6% acetic acid vinegar recovered water quantity performance and mechanical properties when the influent water contained bacteria only but damaged fibers in the presence of sediment. Lastly, water was an acceptable alternative to backwash UF membranes to recover mechanical properties when fibers were fouled with influent water containing humic acid, calcium, and/or sediments. 

The limitations of this work include: (1) filtration and backwashing were performed on small modules with a few membrane fibers instead of full-size filters; (2) in this 10-day study, *E. coli* breakthrough was not reached, and, therefore, the research could not determine whether fouling development or backwashing was the reason for *E. coli* breakthroughs seen in the field; (3) a constant low-pressure backwash was applied to constantly wetted fibers, which may not mimic field conditions where pressure could be variable and fibers could dry out; and, (4) some orange deposition was noted in vinegar-cleaned modules with BacSed influent water (potentially iron deposition, but more investigation is needed). Additionally, some parameters (including fiber material properties) were outside the scope of this study and could be responsible for the differences between membrane types and influent waters observed after filtration and backwash. Therefore, detailed mechanisms behind the observations could be further investigated. One reason why these parameters were not investigated is that companies manufacturing HFMFs for sale in LMICs and humanitarian contexts change the membranes inside their cartridges without notice; hence, this information may not be clear. Despite these limitations, the results from this large exploratory study show that fouling develops rapidly (<200 h of use) on fibers, depending on HFMF type and influent water quality, and that there is no one-size-fits-all backwashing solution. Further laboratory testing should include confirmatory testing of full-scale filters to confirm the results presented herein.

This work highlights the need for future research on fouling in HFMF filters for HWT. Additional laboratory work should focus on expanding testing with other field-relevant influent water qualities but also to test additional cleaning solutions (including other available and easy-to-use concentrations) and backwashing solution combinations (including soaking and the consecutive use of two different cleaning solutions, which could be more effective, although more difficult for the user) [22,41,42]. Reaching *E. coli* breakthrough by changing filtration and backwash conditions should also be considered (including the timeframe of experiment and backwashing pressure), as well as the performance when fibers continually go through wet–dry phases. Lastly, completing field studies seem necessary to determine if cleaning methods can be adopted but also to measure water quality and fouling indicators in collaboration with users over time. Results could help develop end-of-life indicators for HFMFs. 

To conclude, based on these exploratory results that provide unique quantitative information on fouling and cleaning mechanisms on HFMFs in LMICs and humanitarian contexts, implementers of HFMFs should understand that all HFMFs will foul, and there are no one-size-fits-all cleaning mechanisms to prevent fouling in HFMFs. Thus, wide-scale distribution of HFMFs for long-term use in contexts where influent water quality is unknown cannot be recommended. This study highlights the importance of characterizing the water composition of the most probable water sources for each context HFMFs will be distributed. This would lead to a better selection of HFMF type and guide membrane cleaning procedures during HFMF distribution programs. Alternatively, organizations distributing HWT options could also consider alternate options, such as chlorination, other filtration options (e.g., ceramic filters), and/or solar disinfection [43]. As also promoted by some commercial suppliers [44], HFMFs can also be distributed as temporary filters, intended to be replaced after three months of use, before *E. coli* breakthrough [13], or until we know the reasons for breakthrough and how to prevent it. 

## Figures and Tables

**Table 1:** Definition of baseline and endline indicators.

Indicator	Definition
Strain at Break [%]	Baseline: Average strain at break for “Base Wet” fiber
	Endline: Average strain at break for fiber at study end
Break Force [mN]	Baseline: Average break force for “Base Wet” fiber
	Endline: Average break force for fiber at study’s end
Normalized Flow	Baseline: Flow measured before initial filtration run.
	Endline: Average flow at Days 7–10 of filtration
*E. coli* [LRV]	Baseline: Average *E. coli* LRV at Days 1–2 of filtration
	Endline: Average *E. coli* LRV at Days 7–10 of filtration
Turbidity [TRV]	Baseline: Average turbidity LRV at Days 1–2 of filtration
	Endline: Average turbidity LRV at Days 7–10 of filtration

## Data Availability

We confirm all data and images are available from the corresponding author upon request.

## Supplementary Materials

The following are available online at https://www.mdpi.com/article/10.3390/membranes11050375/s1, Figure S1: Example modules; Figure S2: Schematic of filtration apparatus setup during (a) effluent collection (overnight), (b) sample collection (morning), and (c) backwashing; Figure S3: Modules drying for postmortem analysis; Equations (S1)–(S6); Table S1: Filtration system characteristics and influent water quality (mean (SD), sample size N) by influent water and filtration unit/filter type; Table S2: Select baseline and all endline indicators (mean (SD), with sample size N) by filter for all modules; Table S3: Influence of dry/wet fibers (mean (SD), with sample size N) by filter; Table S4: Fouling confirmation for nonbackwashed control modules between baseline and endline (mean (SD), with sample size N) by filter type; Table S5: Fouling consequences for nonbackwashed control modules (mean (SD), with sample size N) by filter type and influent water at endline; Table S6: Filter performance indicators for nonbackwashed (C, control) and backwashed (P, all cleaning methods) modules (mean (SD), with sample size N), stratified by filter type and influent water condition; Table S7: Filter performance indicators for backwashed modules at endline (mean (SD), with sample size N), stratified by filter type, influent water, and cleaning solution (water, bleach, and vinegar); Table S8: Fouling removal indicators for backwashed modules at endline (mean (SD), with sample size N), stratified by filter type, influent water, cleaning solution (water, bleach, and vinegar), and completion of last backwash; Figure S4: Normalized flow at endline by filter type, influent water condition and cleaning solution; Figure S5: *E. coli* LRV at endline by filter type, influent water condition, and cleaning solution; Figure S6: Turbidity reduction at endline by filter type, influent water condition, and cleaning solution; Figure S7: Strain at break at endline by filter type, influent water condition, (last) backwashing completion, and cleaning solution; Figure S8: Break force by filter type, influent water condition, (last) backwashing completion, and cleaning solution; Figure S9: SEM images of fouling development on control fibers for F1–F4 by influent waters; Figure S10: SEM images of fouling control on F1 fibers by cleaning solutions; Figure S11: SEM images of fouling control on F2 fibers by cleaning solutions; Figure S12: SEM images of fouling control on F3 fibers by cleaning solutions; Figure S13: SEM images of fouling control on F4 fibers by cleaning solutions.
