# Supplementary material for: Laboratory Efficacy of Locally Available Backwashing Methods at Removing Fouling in Hollow-Fiber Membrane Filters Used for Household Water Treatment
Source: Membranes (Basel). 2021 May 20;11(5):375. doi: 10.3390/membranes11050375 (PMC8161404; doi:10.3390/membranes11050375)
Supplement: Supplementary file 1 [file membranes-11-00375-s001.zip › membranes-1178349-supplementary.pdf]

# SUPPORTING INFORMATION

# Laboratory Efficacy of Locally Available Backwashing Methods at Removing Fouling in Hollow-Fiber Membrane Filters Used for Household Water Treatment

**Camille Heylen [1,*], Alice Oliveira Aguiar [2], Gabrielle String [1], Marta Domini [1], Nathaniel Goff [2], Anna Murray [1], Ayse Asatekin [2] and Daniele Lantagne [1]**

[1] Department of Civil and Environmental Engineering, Tufts University, Medford, MA 02155, USA; gabrielle.string@tufts.edu (G.S.); marta.domini@unibs.it (M.D.); zutana@gmail.com (A.M.); daniele.lantagne@tufts.edu (D.L.)
[2] Department of Chemical and Biological Engineering, Tufts University, Medford, MA 02155, USA; aliceoa00@gmail.com (A.O.A.); nathaniel.goff@tufts.edu (N.G.); ayse.asatekin@tufts.edu (A.A.)
* Correspondence: camille.heylen@tufts.edu

## Additional Methods Details

*Preparation of modules for testing.*

Each module (Figure S1) corresponds to a number of individual fibers potted in a U-shape (F1-3) or straight (F4) into ¼" internal diameter semi-clear, crack-resistant polyethylene tubes, and glued to create a tight seal without blocking fiber pores.

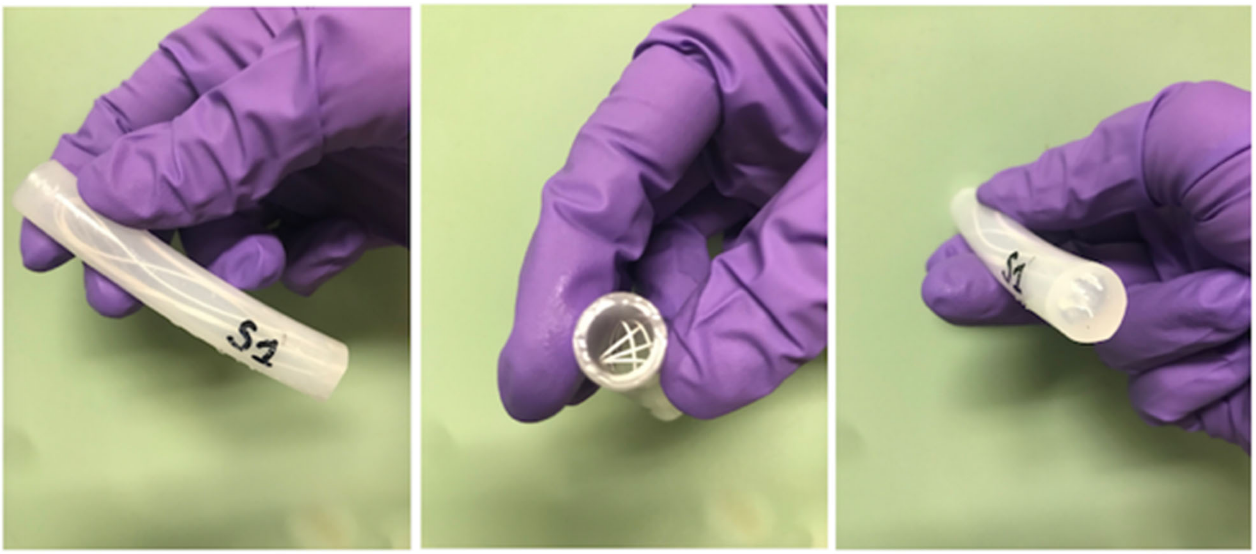


**Figure S1. Example modules.** Pictured here showing the influent end (center) and the effluent end (right) glued in place.

*Filtration and backwashing systems*

The filtration apparatus was composed by four filtration units, each one dedicated to the same filter type over the three runs (Figure S2). Each unit consisted of an impervious plastic 20-L tank on a stir plate, connected to eight modules. Air valve, regulator and, pressure gauge controlled the transmembrane pressure. Modules were placed through a rubber element to tight the system when placed in a 2L-bucket during the first 14 hours of filtration to collect the effluent (Figure S2a). Every day, during the morning filtration, modules were placed into a sterile 118 mL WhirlPak™ bags for E.coli, turbidity and flow testing (FigureS2b). Backwash unit consisted of three 1-L tanks filled with cleaning solution, connected to four modules via 1/4" ID pipes, and an air regulator and pressure gauge (Figure S2c).

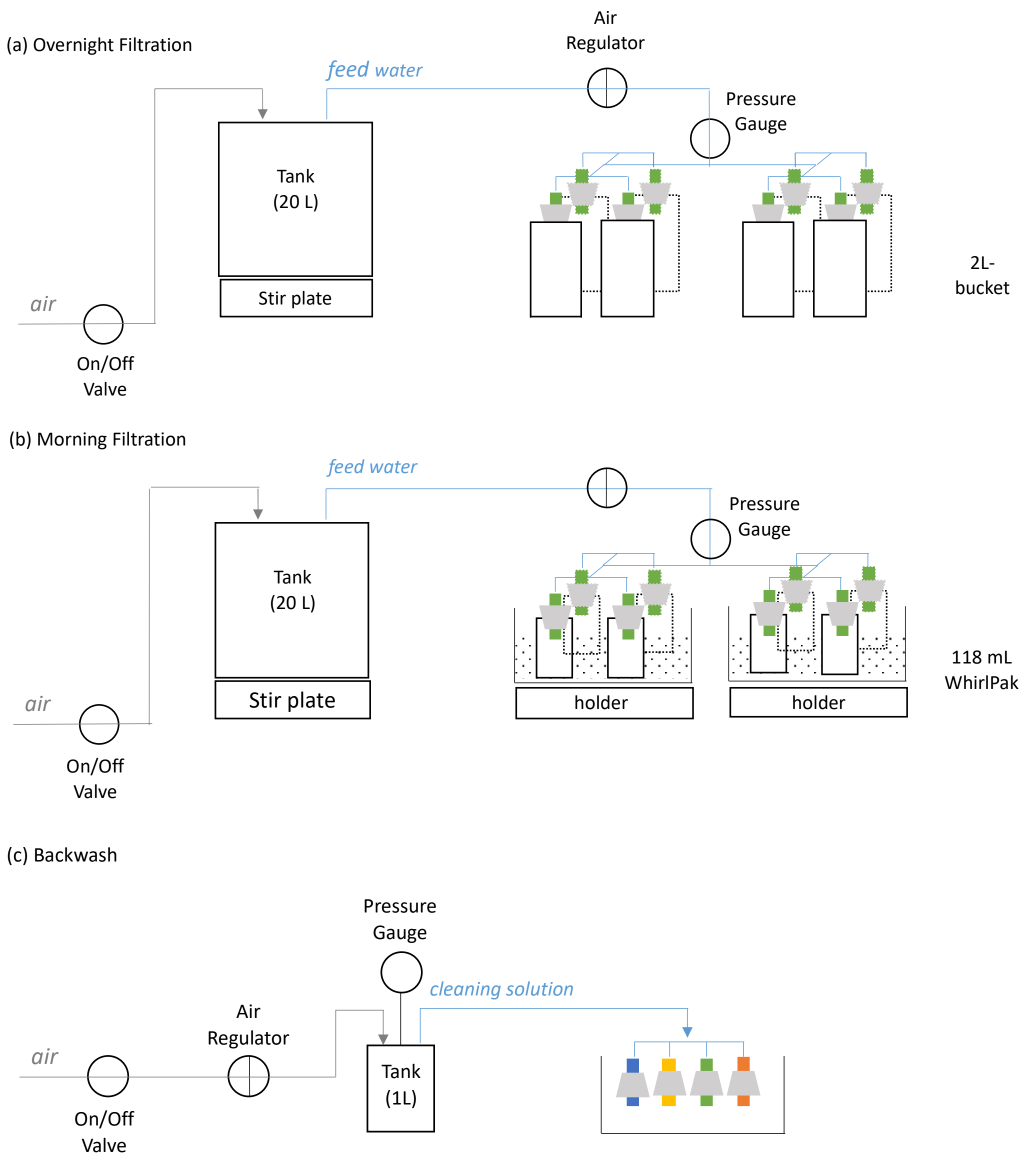


**Figure S2. Schematic of filtration apparatus setup during (a) effluent collection (overnight), (b) sample collection (morning) and, (c) backwashing.** Please note, this graphic only depicts one example apparatus setup. In practice there were four overnight and morning filtration setups (one each for F1-F4) and three backwash apparatuses (one for each backwashing fluid).

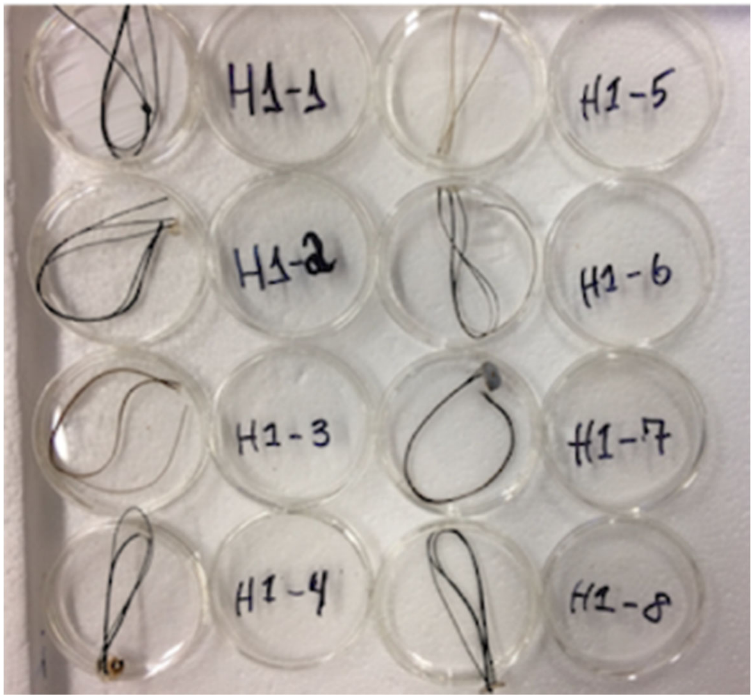


**Figure S3. Modules drying for post-mortem analysis.** Please note, visible fouling on the fibers in this image resulting from the BacSed influent water condition.

*Description of the indicators used to assess fouling development and control*

Strain at break

The strain at break [%] is the ratio of the change in length of the fiber at the breaking point by the initial length of the fiber and is defined by:

$$Strain\ at\ break = \frac{(L - L_0)}{L_0}.100 \qquad \text{(Eq. S1)}$$

with $L_0$ [m] and $L$ [m] the initial length of the fiber and the length at break, respectively.

Break force

The break force $F$ [mN] is the amount of force applied to the sample at its breaking point and directly measured by the tensile test.

Normalized flow

The flow [$L.h^{-1}.bar^{-1}$] at time $t$ [s] was calculated as:

$$Q(t) = \frac{w}{\rho.t.P} \qquad \text{(Eq. S2)}$$

where $w$ [kg] is the weight of the collected water for each sample , ρ [$kg \cdot L^{-1}$] is the density of water fixed to the constant 998 $kg \cdot L^{-1}$ (room temperature of 20°C), $t$ is the time to collect the sample [h] and, $P$ [bar] is the trans-membrane pressure applied on the system. The flows measured during the “second” filtration were averaged from day 7 to day 10 when values showed a plateau, and divided by the initial flow to obtain a normalized flow [-]:

$$\bar{Q}(t) = \frac{Q(t)}{Q(t=0)} \qquad \text{(Eq. S3)}$$

E.coli log reduction value

*E.coli* log reduction value *LRV* [-] was calculated as the logarithm of the ratio between $C_{eff}$ [CFU/100 mL] and $C_{inf}$ [CFU/100 mL] which are the geometric mean of *E. coli* concentration of the effluent and influent, respectively:

$$LRV = -\log\left(\frac{C_{eff}}{C_{inf}}\right) \quad \text{(Eq. S4)}$$

Turbidity reduction value

The turbidity reduction value (*TRV*) [%] is given by the ratio of the difference between influent and effluent turbidity, and influent turbidity, respectively:

$$TRV = \frac{(T_{inf} - T_{eff})}{T_{inf}}.100 \quad \text{(Eq. S5)}$$

Turbidity value, expressed in NTU, is the average of the triplicate results. Negative turbidity reduction values were dropped for the analysis of the results.

Relative change

The relative change [%] for a given indicator was calculated as the ratio of the difference between baseline and endline (after 10 days of filtration) performance, and baseline performance:

$$Relative\ change = \frac{(I_{baseline} - I_{endline})}{I_{baseline}} \quad \text{(Eq. S6)}$$

Baseline and endline definitions were dependent on the indicator, and endline values were averaged over the days after a plateau was reached (Table S1). For mechanical properties, "Base Wet" fibers were used as baseline for both control and backwashed modules, and post-mortem fibers were considered for endline. To quantify changes in water quantity performance, the initial flow before filtration and the averaged flow from Days 7-10 were used as baseline and endline, respectively. Lastly, to measure changes in water quality performance, the *E. coli* LRV and TRV values from the first two days were averaged and compared to values from Days 7-10 of filtration. Note that by measuring relative change in water quality performance in this way, we assumed that fouling or cleaning regimen impacts were negligeable during the first two days of filtration.

## Additional Results

**Table S1. Filtration system characteristics and influent water quality (mean (SD), sample size N) by influent water and filtration unit/filter type.**

| Filtration system parameters | | Filtration Unit | | | |
|---|---|---|---|---|---|
| | | F1<br>(*N=10*) | F2<br>(*N=10*) | F3<br>(*N=10*) | F4<br>(*N=10*) |
| Bacteria | *E. coli* ($10^6$ CFU/100 mL) | 0.91(0.93) | 1.1 (1.4) | 1.0 (1.4) | 0.97 (0.80) |
| | Turbidity (NTU) | 0.10 (0.090) | 0.13 (0.12) | 0.090 (0.090) | 0.090 (0.070) |
| | TDS (mg/L) | 460 (15.5) | 425 (9.80) | 426 (10.2) | 428 (9.70) |
| | pH (-) | 6.8 (0.05) | 6.7 (0.04) | 6.7 (0.05) | 6.7 (0.03) |
| | Time of filtration (hours) | 18.9 (2.0) | 18.7 (2.0) | 19.1 (1.8) | 18.8 (1.9) |
| | Applied pressure (psi) | 0.69 (0.10) | 0.69 (0.10) | 0.69 (0.10) | 0.63 (0.06) |
| BacChem | *E. coli* ($10^6$ CFU/100 mL) | 1.6 (1.0) | 1.0 (0.6) | 1.6 (1.0) | 1.2 (0.8) |
| | Humic Acid weight (g) | 0.63 (0.004) | 0.63 (0.005) | 0.63 (0.006) | 0.63 (0.005) |
| | $CaCl_2$ weight (g) | 1.1 (0.005) | 1.1 (0.005) | 1.1 (0.006) | 1.1 (0.008) |
| | Turbidity (NTU) | 4.5 (0.24) | 4.5 (0.32) | 4.7 (0.33) | 4.6 (0.24) |
| | TDS (mg/L) | 554 (54.6) | 498 (11.4) | 530 (54.2) | 503 (10.8) |
| | pH (-) | 6.8 (0.07) | 6.7 (0.04) | 6.7 (0.05) | 6.7 (0.03) |
| | Time of filtration (hours) | 20.5 (1.5) | 20.5 (1.5) | 20.4 (1.8) | 20.2 (1.8) |
| | Applied pressure (psi) | 0.89 (0.04) | 0.88 (0.06) | 0.90 (0.09) | 0.84 (0.04) |
| BacSed | *E. coli* ($10^6$ CFU/100 mL) | 0.93 (0.52) | 1.4 (1.6) | 1.4 (1.5) | 1.2 (1.1) |
| | Dust weight (g) | 2.2 (0.087) | 2.2 (0.088) | 2.2 (0.086) | 2.2 (0.085) |
| | Turbidity (NTU) | 26 (3.1) | 27 (3.9) | 28 (4.6) | 28 (4.8) |
| | TDS (mg/L) | 464 (47.7) | 435 (24.2) | 437 (15.0) | 434 (16.2) |
| | pH (-) | 6.8 (0.03) | 6.8 (0.02) | 6.7 (0.02) | 6.7 (0.03) |
| | Time of filtration (hours) | 20.0 (1.5) | 20.1 (1.5) | 19.9 (1.6) | 19.8 (1.7) |
| | Applied pressure (psi) | 0.78 (0.01) | 0.78 (0.01) | 0.78 (0.01) | 0.74 (0.01) |

**Table S2. Select baseline and all endline indicators (mean (SD) with sample size N) by filter for all modules. P-values from Kruskal-Wallis tests.**

| | | Filter | | | | p-value | p-value | p-value |
|---|---|---|---|---|---|---|---|---|
| | | F1 | F2 | F3 | F4 | (F1-F4) | (F1-F3) | (F1-3$_{avg}$ to F4) |
| Select baseline indicators | Strain at break (Base Dry) % | 27 (4.3) (N=4) | 28 (0.7) (N=4) | 29 (2.1) (N=4) | 51 (4.9) (N=4) | *** (p=0.031)** | NS (p=0.69) | **** (p=0.003)** |
| | Strain at break (Base Wet) *%* | 30 (3.7) (N=8) | 31 (1.5) (N=8) | 32 (1.8) (N=8) | 46 (1.6) (N=8) | ***** (p<0.001)** | NS (p=0.49) | ***** (p<0.001)** |
| | Break force (Base Dry) mN | 300 (8.2) (N=4) | 331 (14.3) (N=4) | 316 (5.0) (N=4) | 1,610 (36.9) (N=4) | **** (p=0.004)** | *** (p=0.012)** | **** (p=0.004)** |
| | Break force (Base Wet) *mN* | 298 (10.3) (N=8) | 329 (7.7) (N=8) | 312 (12.5) (N=8) | 1,827 (49.9) (N=8) | ***** (p<0.001)** | ***** (p<0.001)** | ***** (p<0.001)** |
| Endline indicators | Normalized Flow | 0.20 (0.30) (N=92) | 0.15 (0.15) (N=88) | 0.16 (0.16) (N=92) | 0.67 (0.36) (N=80) | ***** (p<0.001)** | NS (p=0.14) | ***** (p<0.001)** |
| | *E. coli* LRV | 5.4 (0.70) (N=125) | 5.3 (0.73) (N=122) | 5.3 (0.69) (N=137) | 5.6 (0.86) (N=128) | ***** (p<0.001)** | NS (p=0.71) | ***** (p<0.001)** |
| | Turbidity *NTU* | 0.58 (0.55) (N=87) | 0.49 (0.46) (N=87) | 0.51 (0.42) (N=93) | 0.45 (0.37) (N=80) | NS (p=0.44) | NS (p=0.56) | NS (p=0.22) |
| | Strain at break % | 15 (8.7) (N=90) | 13 (7.3) (N=85) | 14 (8.0) (N=92) | 24 (7.1) (N=92) | ***** (p<0.001)** | NS (p=0.43) | ***** (p<0.001)** |
| | Break force *mN* | 274 (25.4) (N=90) | 289 (27.7) (N=85) | 289 (31.0) (N=92) | 1,716 (112) (N=92) | ***** (p<0.001)** | ***** (p<0.001)** | ***** (p<0.001)** |

NS ($p>0.05$), * ($p \leq 0.05$),**($p \leq 0.01$), ***($p \leq 0.001$)
The initial strain at break and break force were determined with fibers without any treatment (Base Dry) and those soaked for 10 days in DI (Base Wet)
The TVR combined values for BacChem and BacSed, and no values for Bacteria taken into account as initial turbidity was too low to measure the TVR.

**Table S3. Influence of dry/wet fibers (mean (SD) with sample size N) by filter. P-values from Kruskal-Wallis tests.**

| | | Filter | | | | | | | |
|---|---|---|---|---|---|---|---|---|---|
| | | F1 | | F2 | | F3 | | F4 | |
| Baseline indicators | Strain at break (Base Dry) % | 27 (4.3) (N=4) | NS (p=0.199) | 28 (0.7) (N=4) | * **(p=0.016)** | 29 (2.1) (N=4) | * **(p=0.028)** | 51 (4.9) (N=4) | NS (p=0.154) |
| | Strain at break (Base Wet) % | 30 (3.7) (N=8) | | 31 (1.5) (N=8) | | 32 (1.8) (N=8) | | 46 (1.6) (N=8) | |
| | Break force (Base Dry) mN | 300 (8.2) (N=4) | NS (p=0.825) | 331 (14.3) (N=4) | NS (p=0.933) | 316 (5.0) (N=4) | NS (p=0.683) | 1,610 (36.9) (N=4) | ** **(p=0.004)** |
| | Break force (Base Wet) mN | 298 (10.3) (N=8) | | 329.4 (7.7) (N=8) | | 312 (12.5) (N=8) | | 1,827 (49.9) (N=8) | |

**Table S4. Fouling confirmation for non-backwashed control modules between baseline and endline (mean (SD) with sample size N) by filter type. P-values from Wilcoxon signed-rank tests.**

| Filter Type | MF membranes (F1-$3_{avg}$) | | | UF membranes (F4) | | |
|---|---|---|---|---|---|---|
| | Baseline | Endline | p-value | Baseline | Endline | p-value |
| Normalized Flow | 1 | 0.07 (0.04) (N=68) | ***** (p<0.001)** | 1 | 0.53 (0.30) (N=24) | ***** (p<0.001)** |
| *E. coli* LRV | 5.1 (0.67) (N=24) | 5.2 (0.86) (N=92) | NS (p=0.61) | 5.2 (0.78) (N=8) | 5.0 (1.66) (N=32) | NS (p=0.818) |
| TRV % | 86 (15) (N=24) | 94 (4.7) (N=60) | NS (p=0.195) | 79 (31) (N=8) | 72 (48) (N=25) | NS (p=0.886) |
| Strain at break % | 28 (2.6) (N=12) | 7.3 (3.8) (N=56) | ***** (p<0.001)** | 51 (4.9) (N=4) | 24 (6.5) (N=28) | ***** (p<0.001)** |
| Break force mN | 316 (16) (N=12) | 261 (25) (N=56) | ***** (p<0.001)** | 1827 (50) (N=4) | 1762 (90) (N=28) | * (p=0.017) |

NS (p>0.05), * (p ≤ 0.05),**(p ≤ 0.01), ***(p ≤ 0.001)

**Table S5. Fouling consequences for non-backwashed control modules (mean (SD) with sample size N) by filter type and influent water at endline. P-values from Kruskal-Wallis tests.**

| Filter Type | MF membranes (F1-$3_{avg}$) | | | UF membranes (F4) | | |
|---|---|---|---|---|---|---|
| Influent water | Bacteria | BacChem | BacSed | Bacteria | BacChem | BacSed |
| Normalized Flow | 0.12 (0.03) (N=24) | 0.02 (0.01) (N=24) ***** (p<0.001)** | 0.07 (0.01) (N=20) | 0.74 (0.08) (N=8) | 0.14 (0.02) (N=8) ***** (p<0.001)** | 0.69 (0.04) (N=8) |
| *E. coli* LRV | 5.4 (1.0) (N=32) | 5.0 (0.4) (N=34) *** (p=0.014)** | 5.1 (0.6) (N=26) | 5.1 (1.4) (N=11) | 5.9 (0.5) (N=11) NS (p=0.23) | 5.4 (1.0) (N=10) |
| TRV % | 9.4 (18) (N=8) | 83 (13) (N=28) ***** (p<0.001)** | 98 (0.9) (N=24) | 17 (33) (N=4) | 85 (9.5) (N=9) ***** (p<0.001)** | 98 (0.9) (N=12) |
| Strain at break % | 8 (1.6) (N=17) | 5 (0.8) (N=23) ***** (p<0.001)** | 10 (5.0) (N=16) | 21 (3.2) (N=8) | 20 (5.9) (N=10) ***** (p<0.001)** | 30 (4.0) (N=10) |
| Break force mN | 269 (22) (N=17) | 241 (17) (N=23) ***** (p<0.001)** | 275 (20) (N=16) | 1,721 (62) (N=8) | 1,828 (103) (N=10) NS (p=0.082) | 1,728 (54) (N=10) |

NS (p>0.05), * (p ≤ 0.05),**(p ≤ 0.01), ***(p ≤ 0.001)

**Table S6. Filter performance indicators for non-backwashed (C, control) and backwashed (P, all cleaning methods) modules (mean (SD) with sample size N) stratified by filter type and influent water condition. P-values from Kruskal-Wallis tests.**

| Filter Type | MF membranes (F1-3$_{avg}$) | | | | | | UF membranes (F4) | | | | | |
|---|---|---|---|---|---|---|---|---|---|---|---|---|
| Influent water | Bacteria | | BacChem | | BacSed | | Bacteria | | BacChem | | BacSed | |
| Cleaning method | C | P | C | P | C | P | C | P | C | P | C | P |
| Normalized Flow | 0.12(0.03) (N=24) | 0.43(0.29) (N=72) | 0.02(0.01) (N=24) | 0.04(0.01) (N=72) | 0.07(0.01) (N=20) | 0.17(0.13) (N=72) | 0.74(0.08) (N=8) | 1.1 (0.16) (N=17) | 0.14(0.02) (N=8) | 0.27 (0.13) (N=16) | 0.69(0.04) (N=8) | 0.82(0.28) (N=24) |
| | ***(p<0.001) | | ***(p<0.001) | | *(p=0.018) | | ***(p<0.001) | | *(p=0.027) | | NS (p=0.164) | |
| *E. coli* LRV | 5.4 (1.0) (N=32) | 5.6 (0.8) (N=93) | 5.0 (0.4) (N=34) | 5.0 (0.4) (N=100) | 5.1 (0.6) (N=26) | 5.6 (0.6) (N=99) | 5.1 (1.4) (N=11) | 5.6 (0.7) (N=28) | 5.9 (0.5) (N=11) | 6.0 (0.4) (N=26) | 5.4 (1.0) (N=10) | 5.6 (0.9) (N=36) |
| | NS (p=0.32) | | NS (p=0.91) | | **(p=0.003) | | NS (p=0.56) | | NS (p=0.79) | | NS ( p=0.82) | |
| TRV % | 9.4 (18) (N=8) | 22 (33) (N=43) | 83 (13) (N=28) | 82 (10) (N=87) | 98 (0.9) (N=24) | 98 (2.0) (N=77) | 17 (33) (N=4) | 3.5 (9.2) (N=7) | 85 (9.5) (N=9) | 82 (7.8) (N=17) | 98 (0.9) (N=12) | 98 (0.9) (N=31) |
| | NS (p=0.44) | | NS (p=0.91) | | NS (p=0.30) | | NS (p=0.56) | | NS (p=0.79) | | NS (p=0.82) | |
| Strain at break % | 8 (1.6) (N=17) | 17 (6.7) (N=71) | 5 (0.8) (N=23) | 13 (9.3) (N=69) | 10 (5.0) (N=24) | 18 (6.5) (N=71) | 21 (3.2) (N=8) | 25 (6.7) (N=24) | 20 (5.9) (N=10) | 17 (5.6) (N=24) | 30 (4.0) (N=10) | 28 (2.5) (N=25) |
| | ***(p<0.001) | | ***(p<0.001) | | ***(p<0.001) | | NS (p=0.16) | | NS (p=0.23) | | NS (p=0.09) | |
| Break force mN | 269 (22) (N=17) | 293 (20) (N=71) | 241 (17) (N=23) | 280 (36) (N=69) | 275 (20) (N=16) | 297 (17) (N=71) | 1,721(62) (N=8) | 1,749(134) (N=24) | 1,828(103) (N=10) | 1,703(136) (N=24) | 1,728(54) (N=10) | 1,680 (76) (N=28) |
| | ***(p<0.001) | | ***(p<0.001) | | ***(p<0.001) | | NS (p=0.69) | | NS (p=0.051) | | NS (p=0.070) | |

NS ($p>0.05$), * ($p \leq 0.05$),**($p \leq 0.01$), ***($p \leq 0.001$)

**Table S7. Filter performance indicators for backwashed modules at endline (mean (SD) with sample size N) stratified by filter type, influent water, and cleaning solution (Water, Bleach and Vinegar). P-values from Kruskal-Wallis tests.**

| Filter Type | Microfilter (F1-3) | | | | | | | | | Ultrafilter (F4) | | | | | | | | |
|---|---|---|---|---|---|---|---|---|---|---|---|---|---|---|---|---|---|---|
| Influent water | Bacteria | | | BacChem | | | BacSed | | | Bacteria | | | BacChem | | | BacSed | | |
| Cleaning solution | W | B | V | W | B | V | W | B | V | W | B | V | W | B | V | W | B | V |
| Normalized flow | 0.18 (0.06) (N=21) | 0.77 (0.30) (N=21) | 0.43 (0.22) (N=21) | 0.04 (0.01) (N=24) | 0.04 (0.01) (N=24) | 0.04 (0.01) (N=24) | 0.20 (0.05) (N=24) | 0.28 (0.11) (N=24) | 0.03 (0.01) (N=24) | 0.98 (0.05) (N=7) | 1.33 (0.05) (N=3) | 1.01 (0.15) (N=7) | - - | 0.39 (0.02) (N=8) | 0.14 (0.03) (N=8) | 0.98 (0.08) (N=8) | 1.04 (0.05) (N=8) | 0.45 (0.10) (N=8) |
| | *** **(p<0.001)** | | | NS (p=0.651) | | | *** **(p<0.001)** | | | * **(p=0.029)** | | | *** **(p<0.001)** | | | *** **(p<0.001)** | | |
| *E. coli* LRV | 5.5 (0.8) (N=33) | 5.7 (0.7) (N=30) | 5.6 (0.8) (N=30) | 5.0 (0.4) (N=34) | 5.0 (0.4) (N=33) | 5.0 (0.5) (N=33) | 5.8 (0.6) (N=38) | 5.9 (0.4) (N=30) | 5.1 (0.6) (N=31) | 5.5 (0.9) (N=12) | 5.7 (0.4) (N=6) | 5.6 (0.7) (N=10) | - - | 6.0 (0.4) (N=13) | 5.9 (0.5) (N=13) | 5.0 (1.0) (N=16) | 6.0 (0.4) (N=10) | 6.0 (0.4) (N=10) |
| | NS (p=0.90) | | | NS (p=0.85) | | | *** **(p<0.001)** | | | NS (p=0.99) | | | NS (p=0.70) | | | ** **(p=0.002)** | | |
| TVR % | 18 (32) (N=14) | 22 (35) (N=15) | 25 (35) (N=14) | 81 (11) (N=31) | 81 (12) (N=28) | 85 (7.7) (N=28) | 98 (1.3) (N=26) | 98 (2.5) (N=26) | 97 (2.0) (N=25) | 8.1 (14) (N=3) | 0 (-) (N=1) | 0 (0) (N=3) | - - | 80 (8.2) (N=10) | 85 (6.9) (N=7) | 98 (1.1) (N=12) | 98 (0.7) (N=10) | 98 (0.9) (N=9) |
| | NS (p=0.91) | | | NS (p=0.29) | | | NS (p=0.39) | | | NS (p=0.51) | | | NS (p=0.28) | | | NS (p=0.55) | | |
| Strain at break % | 10 (1.9) (N=23) | 21 (5.4) (N=24) | 19 (5.5) (N=24) | 15 (11) (N=24) | 15 (10) (N=22) | 10 (3.7) (N=23) | 18 (5.1) (N=24) | 25 (2.8) (N=23) | 12 (2.4) (N=24) | 28 (4.4) (N=8) | 17 (1.8) (N=8) | 29 (3.6) (N=8) | 22 (3.9) (N=8) | 19 (4.0) (N=8) | 11 (2.5) (N=8) | 32 (4.5) (N=10) | 26 (2.4) (N=9) | 26 (0.8) (N=9) |
| | *** **(p<0.001)** | | | (p=0.78) | | | *** **(p<0.001)** | | | *** **(p<0.001)** | | | *** **(p<0.001)** | | | *** **(p<0.001)** | | |
| Break force mN | 270 (12) (N=23) | 299 (14) (N=24) | 309 (9.5) (N=24) | 277 (41) (N=24) | 274 (39) (N=22) | 290 (24) (N=23) | 301 (17) (N=24) | 304 (13) (N=23) | 285 (16) (N=24) | 1,727 (141) (N=8) | 1,644 (57) (N=8) | 1,876 (67) (N=8) | 1,829 (102) (N=8) | 1,556 (55) (N=8) | 1,725 (64) (N=8) | 1,765 (38) (N=10) | 1,647 (41) (N=9) | 1,619 (43) (N=9) |
| | *** **(p<0.001)** | | | (p=0.37) | | | *** **(p<0.001)** | | | **(p=0.005)** | | | *** **(p<0.001)** | | | *** **(p<0.001)** | | |

NS (p>0.05), * (p ≤ 0.05),**(p ≤ 0.01), ***(p ≤ 0.001)

**Table S8. Fouling removal indicators* for backwashed modules at endline (mean (SD) with sample size N) stratified by filter type, influent water, cleaning solution (Water, Bleach and Vinegar), and completion of last backwash**

| Filter Type | Influent water | Chemical cleaning method | Last backwash | Strain at break % | Break force mN |
|---|---|---|---|---|---|
| Microfilter (F1-3) | Bacteria | W | No | 9 (1.6) (N=11) | 270 (13) (N=11) |
| | | | Yes | 11 (1.5) (N=12) | 280 (12) (N=12) |
| | | B | No | 17 (3.1) (N=12) | 291 (11) (N=12) |
| | | | Yes | 25 (3.7) (N=12) | 306 (12) (N=12) |
| | | V | No | 17 (5.7) (N=12) | 309 (9) (N=12) |
| | | | Yes | 21 (4.6) (N=12) | 310 (10) (N=12) |
| | BacChem | W | No | 4 (0.6) (N=12) | 239 (11) (N=12) |
| | | | Yes | 25 (6.5) (N=12) | 315 (18) (N=12) |
| | | B | No | 5 (0.9) (N=11) | 237 (10) (N=11) |
| | | | Yes | 25 (2.7) (N=11) | 310 (13) (N=11) |
| | | V | No | 8 (2.3) (N=12) | 274 (15) (N=12) |
| | | | Yes | 13 (3.7) (N=11) | 307 (19) (N=11) |
| | BacSed | W | No | 19 (5.9) (N=12) | 305 (21) (N=12) |
| | | | Yes | 18 (4.5) (N=12) | 298 (14) (N=12) |
| | | B | No | 26 (1.7) (N=12) | 302 (14) (N=12) |
| | | | Yes | 23 (3.1) (N=11) | 306 (11) (N=11) |
| | | V | No | 10 (1.4) (N=12) | 281 (18) (N=12) |
| | | | Yes | 13 (2.2) (N=12) | 289 (13) (N=12) |
| Ultrafilter (F4) | Bacteria | W | No | 25 (2.5) (N=4) | 1599 (31) (N=4) |
| | | | Yes | 31 (4.2) (N=4) | 1855 (37) (N=4) |
| | | B | No | 17 (2.2) (N=4) | 1615 (47) (N=4) |
| | | | Yes | - | - |
| | | V | No | 30 (2.3) (N=4) | 1895 (57) (N=4) |
| | | | Yes | 28 (4.7) (N=4) | 1856 (79) (N=4) |
| | BacChem | W | No | - | - |
| | | | Yes | - | - |
| | | B | No | 15 (1.9) (N=4) | 1540 (76) (N=4) |
| | | | Yes | 22 (0.8) (N=4) | 1572 (21) (N=4) |
| | | V | No | 10 (1.5) (N=4) | 1707 (47) (N=4) |
| | | | Yes | 13 (2.4) (N=4) | 1742 (80) (N=4) |
| | BacSed | W | No | 32 (4.2) (N=5) | 1756 (46) (N=5) |
| | | | Yes | 33 (5.2) (N=5) | 1775 (30) (N=5) |
| | | B | No | 26 (1.7) (N=5) | 1647 (32) (N=5) |
| | | | Yes | 26 (3.4) (N=4) | 1647 (55) (N=4) |
| | | V | No | 26 (0.2) (N=5) | 1591 (7) (N=5) |
| | | | Yes | 27 (1.3) (N=4) | 1653 (46) (N=4) |

* Flow, E. coli and turbidity were not measured after the final study-end backwash (on the $10^{th}$ day), thus we were not able to assess the impact of the final backwash on these indicators.

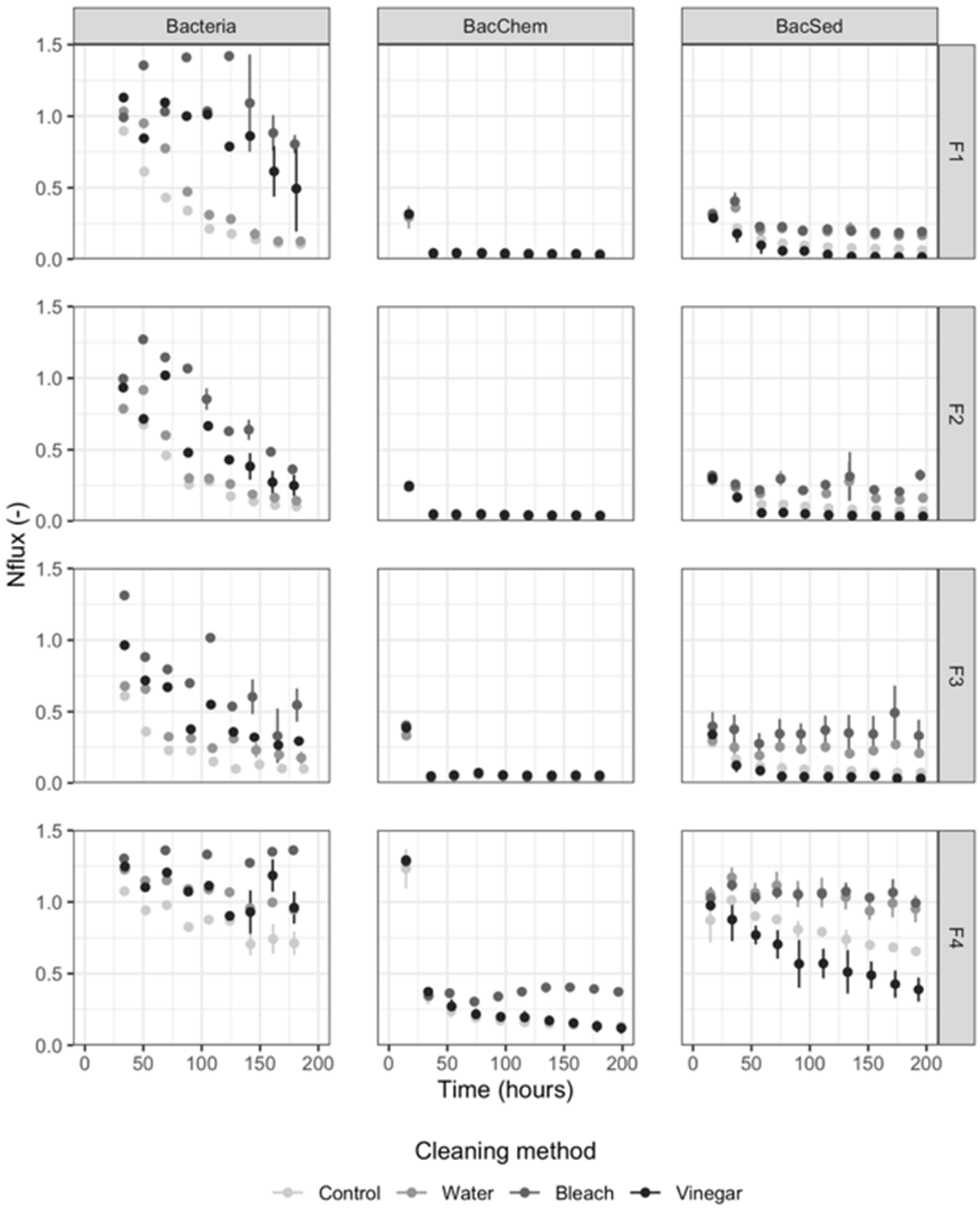


**Figure S4. Normalized flow at endline by filter type, influent water condition and cleaning solution**

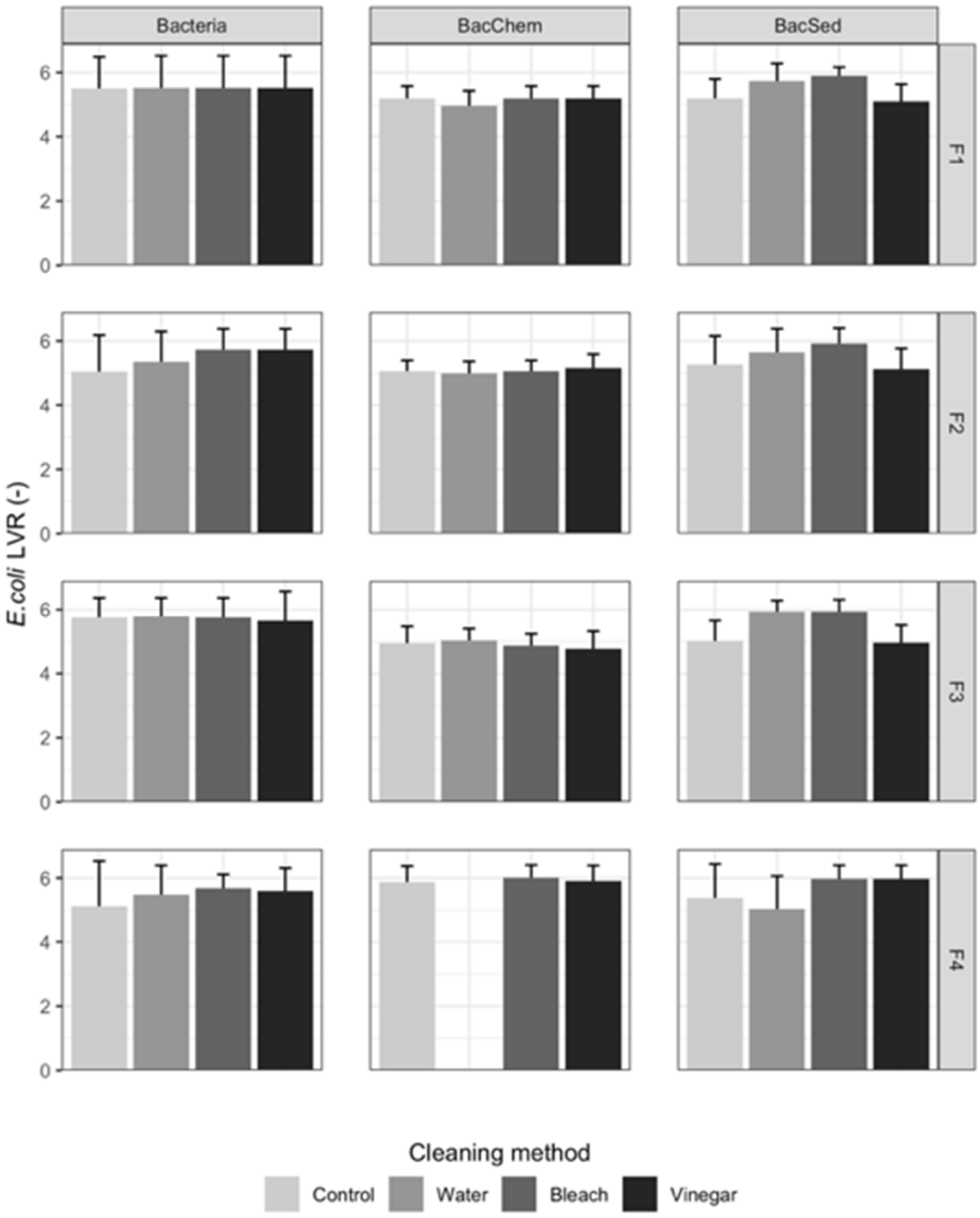


**Figure S5.** ***E. coli*** **LRV at endline by filter type, influent water condition and cleaning solution**

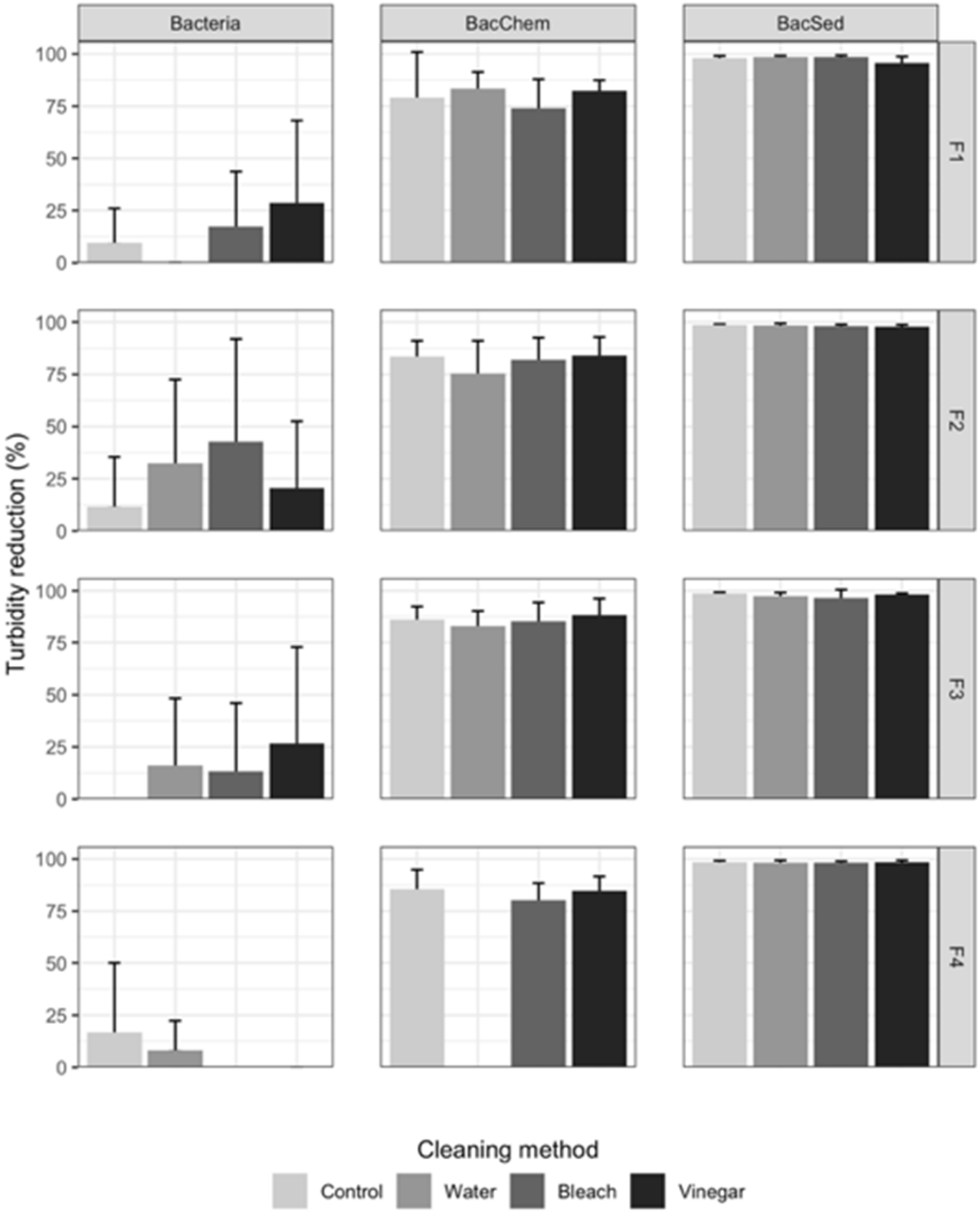


**Figure S6. Turbidity reduction at endline by filter type, influent water condition and cleaning solution**

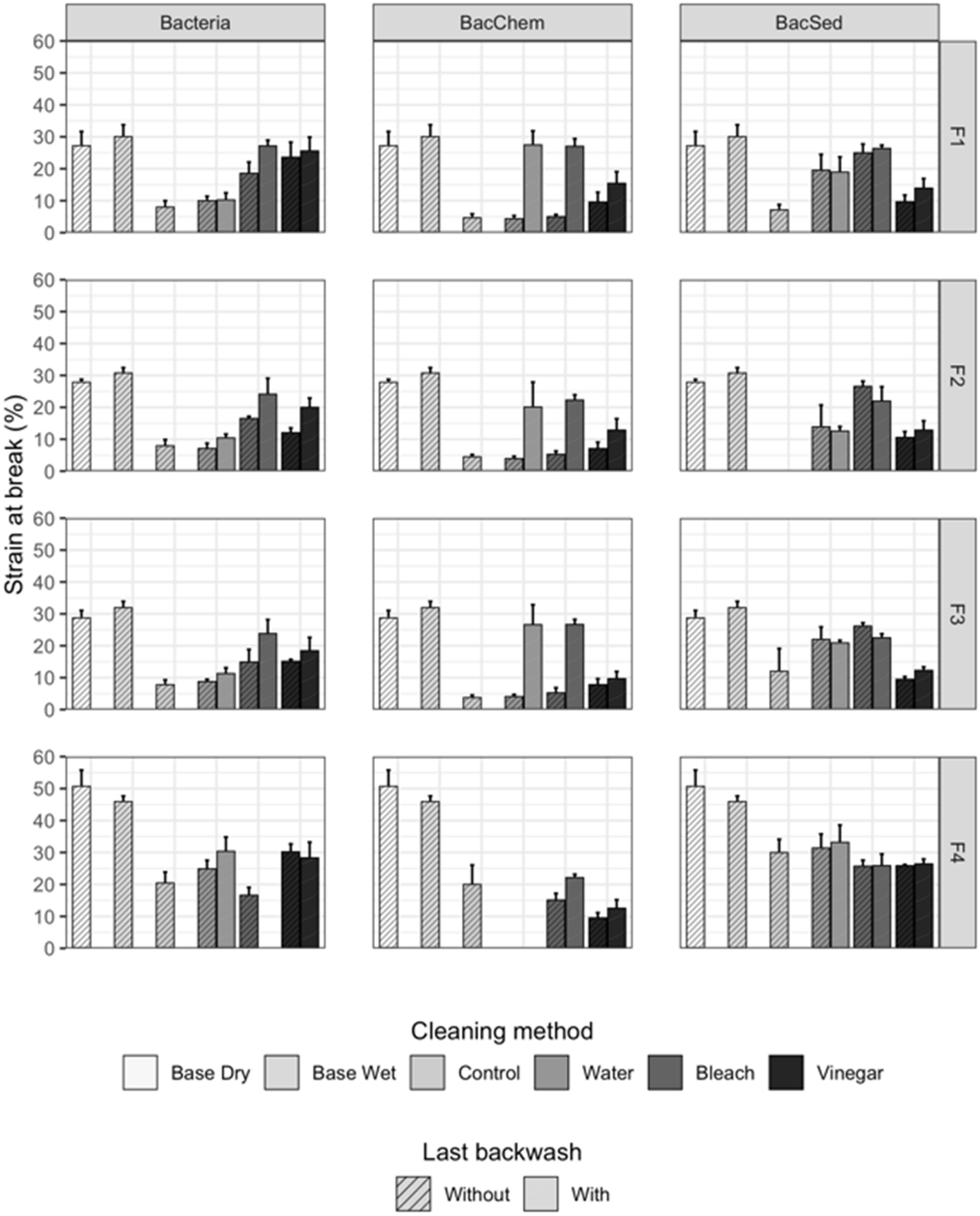


**Figure S7. Strain at break at endline by filter type, influent water condition, (last) backwashing completion and cleaning solution**

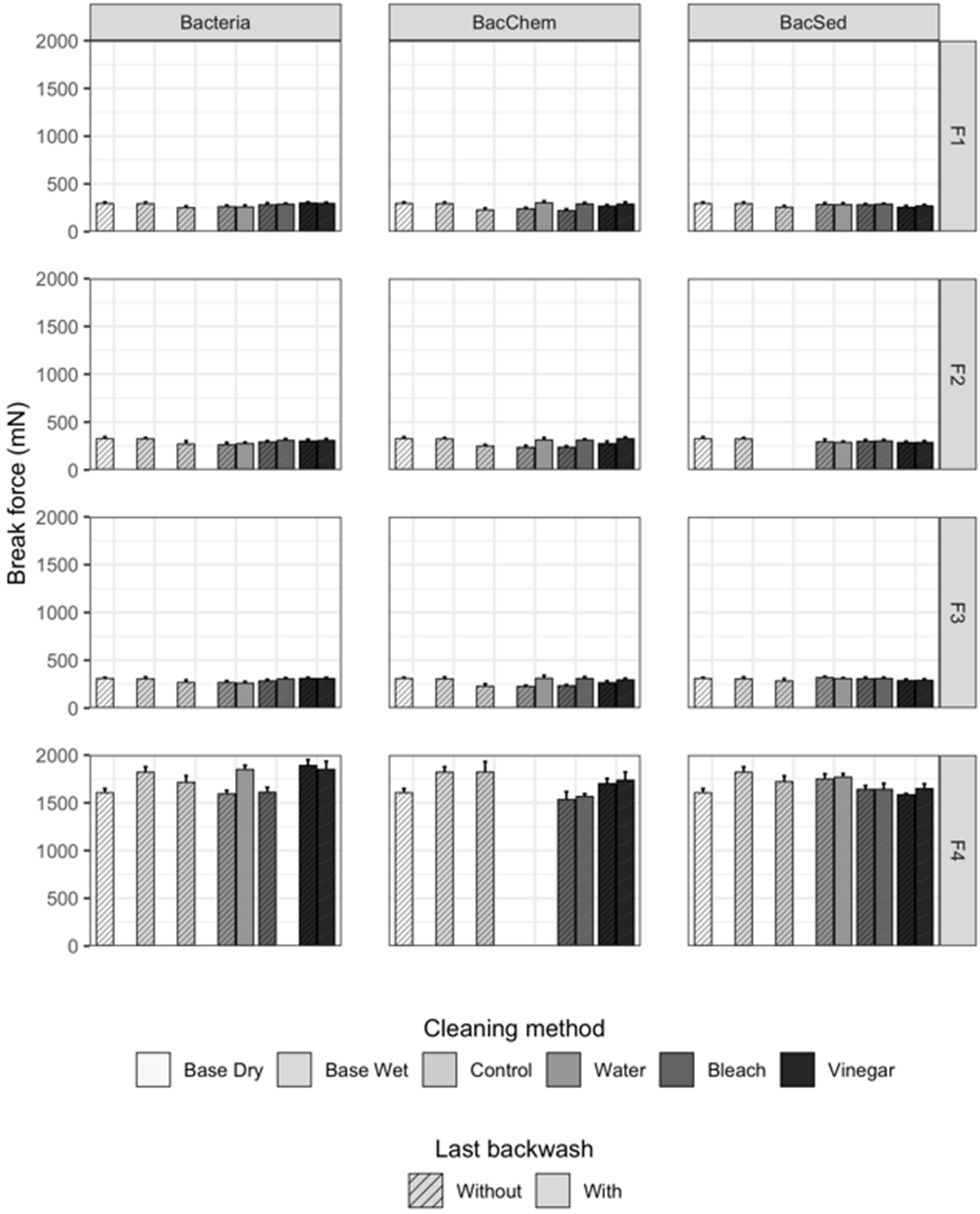


**Figure S8. Break force by filter type, influent water condition, (last) backwashing completion and cleaning solution**

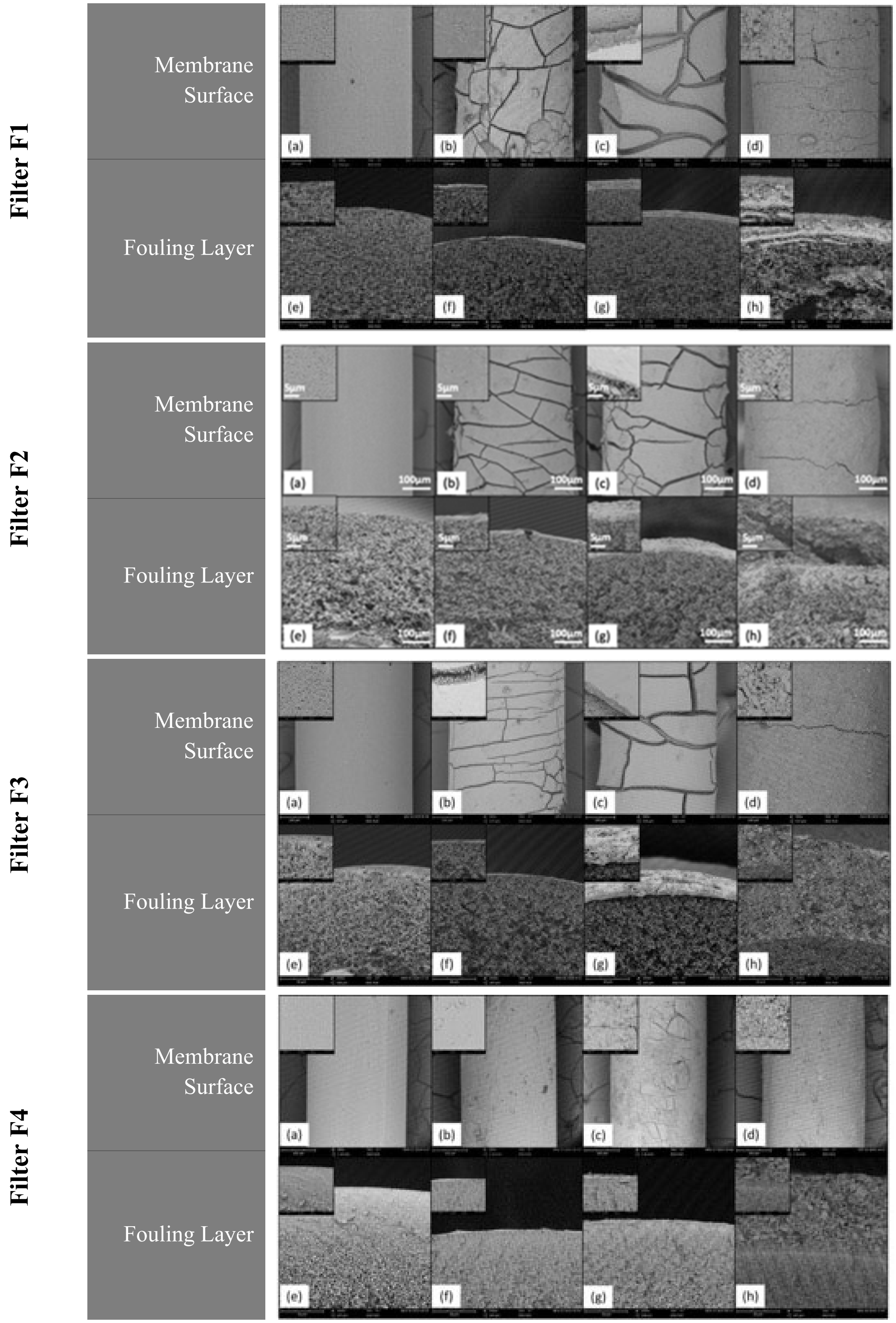


**Figure S9. SEM images of fouling development on control fibers for F1-F4 by influent waters.** Outer membrane surface at 500x and 15,000x (insets) for control: a) "Base Dry" fiber, b) Bacteria, c) BacChem, d) BacSed. Cross-section showing fouling layer thickness on outer surface (2,500x and 10,000x (insets)) for: e) "Base Dry" fiber, f) Bacteria, g) BacChem, h) BacSed.

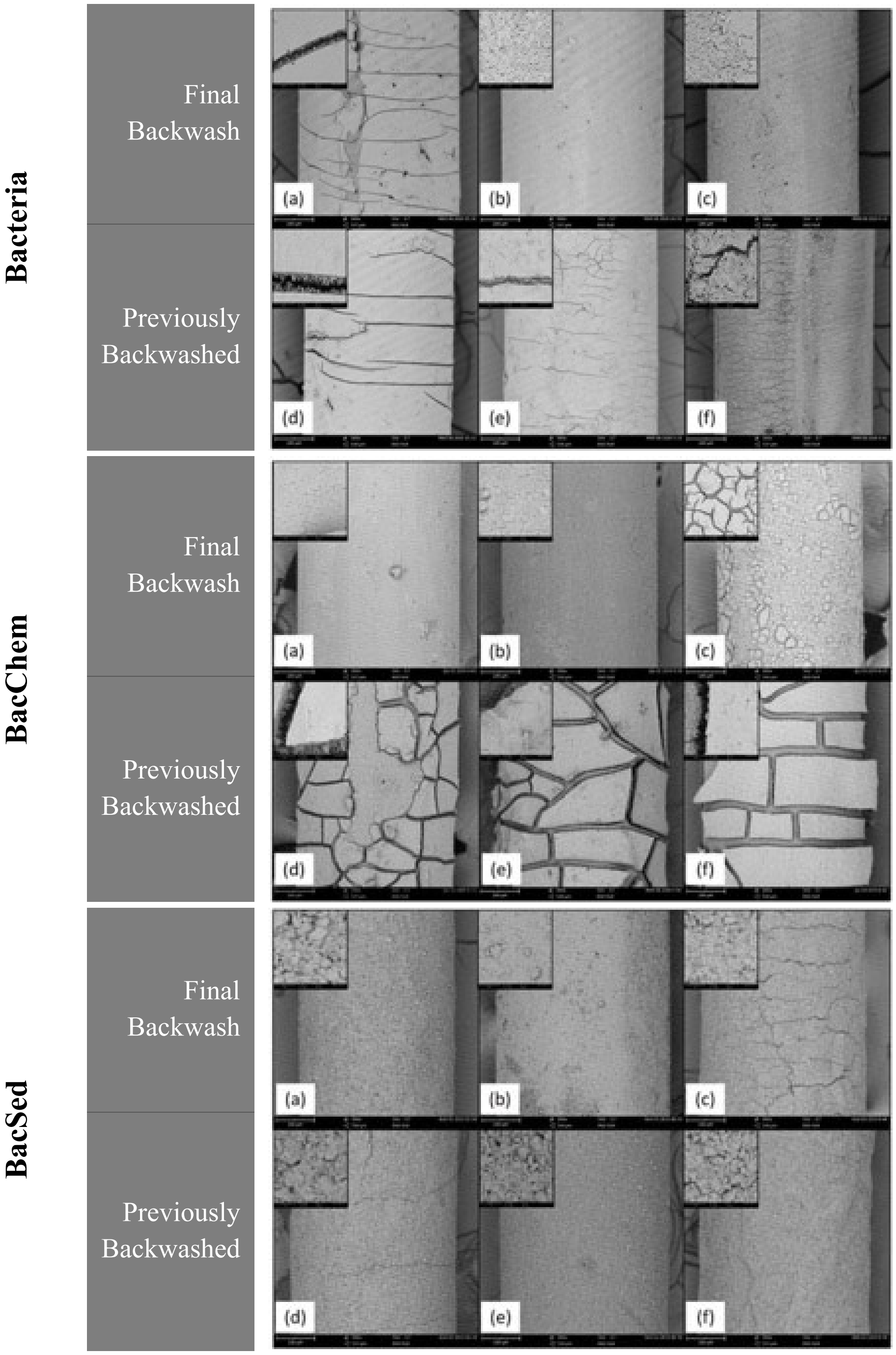


**Figure S10. SEM images of fouling control on F1 fibers by cleaning solutions**. Outer membrane surface at 500x and 15,000x (insets) showing fouling layer after filtration with final backwash with: (a) DI water, (b) bleach, (c) vinegar; without final backwash, but previously backwashed with: (d) DI water, (e) bleach, (f) vinegar.

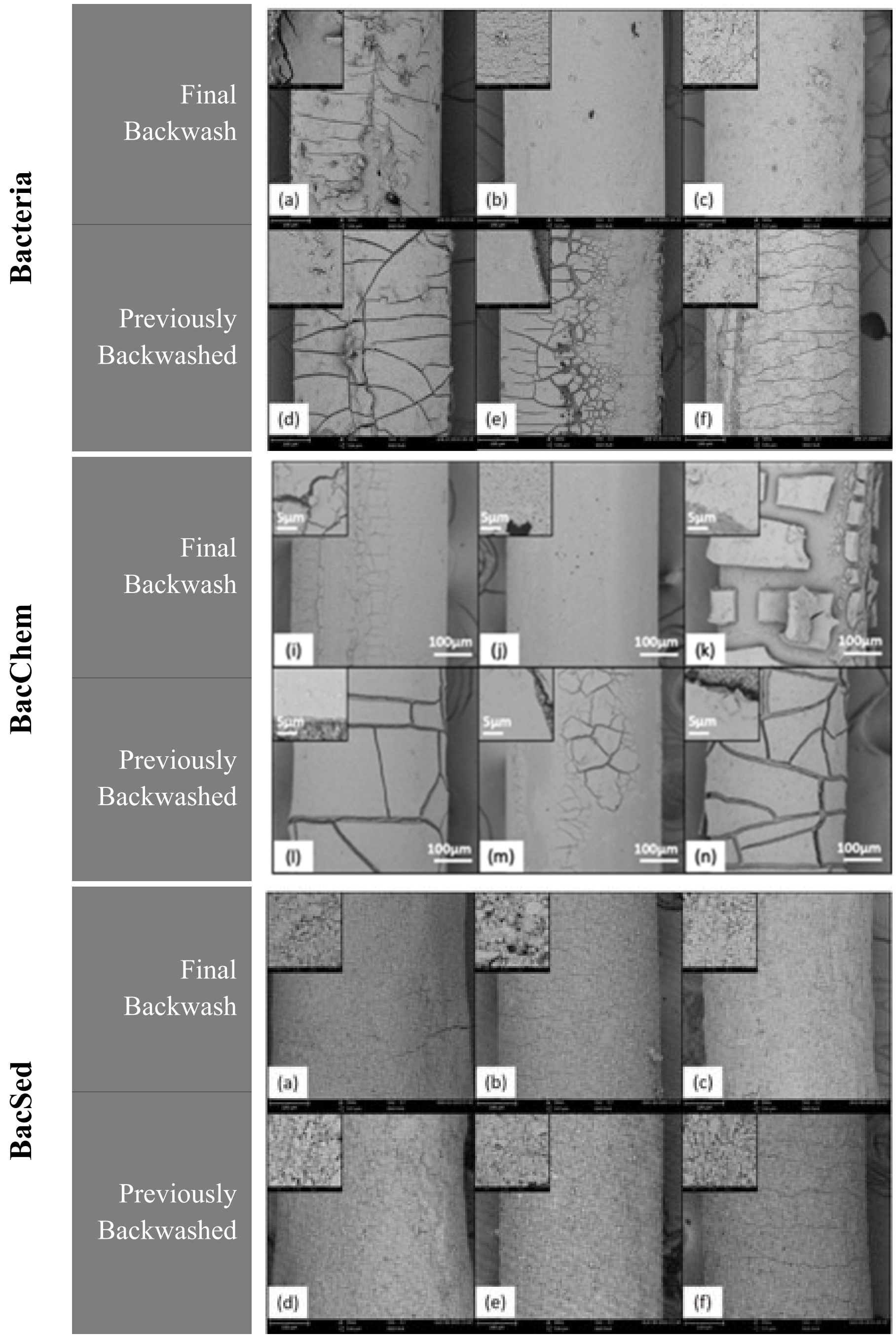


**Figure S11. SEM images of fouling control on F2 fibers by cleaning solutions**. Outer membrane surface at 500x and 15,000x (insets) showing fouling layer after filtration with final backwash with: (a) DI water, (b) bleach, (c) vinegar; without final backwash, but previously backwashed with: (d) DI water, (e) bleach, (f) vinegar.

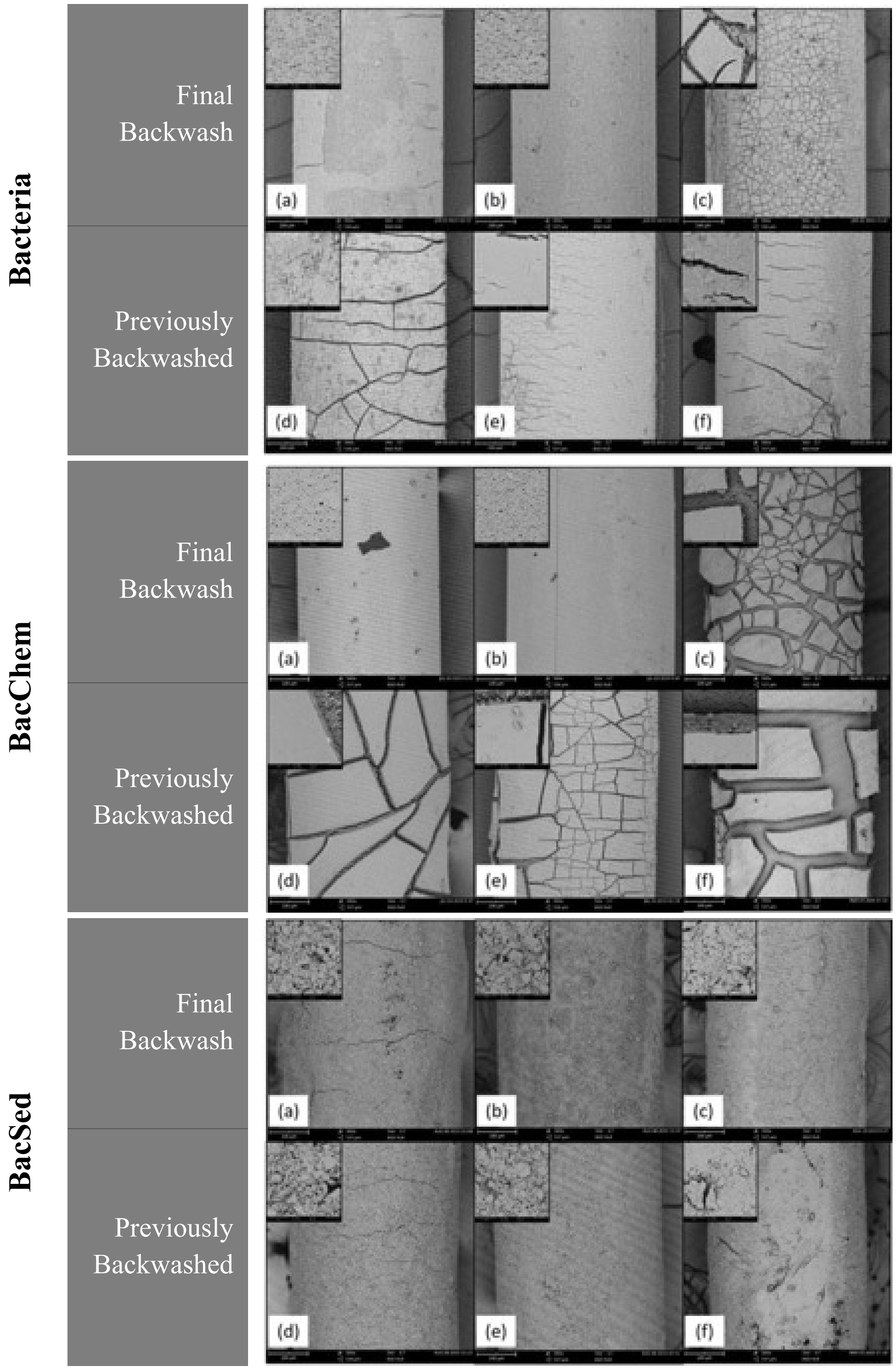


**Figure S12. SEM images of fouling control on F3 fibers by cleaning solutions**. Outer membrane surface at 500x and 15,000x (insets) showing fouling layer after filtration with final backwash with: (a) DI water, (b) bleach, (c) vinegar; without final backwash, but previously backwashed with: (d) DI water, (e) bleach, (f) vinegar.

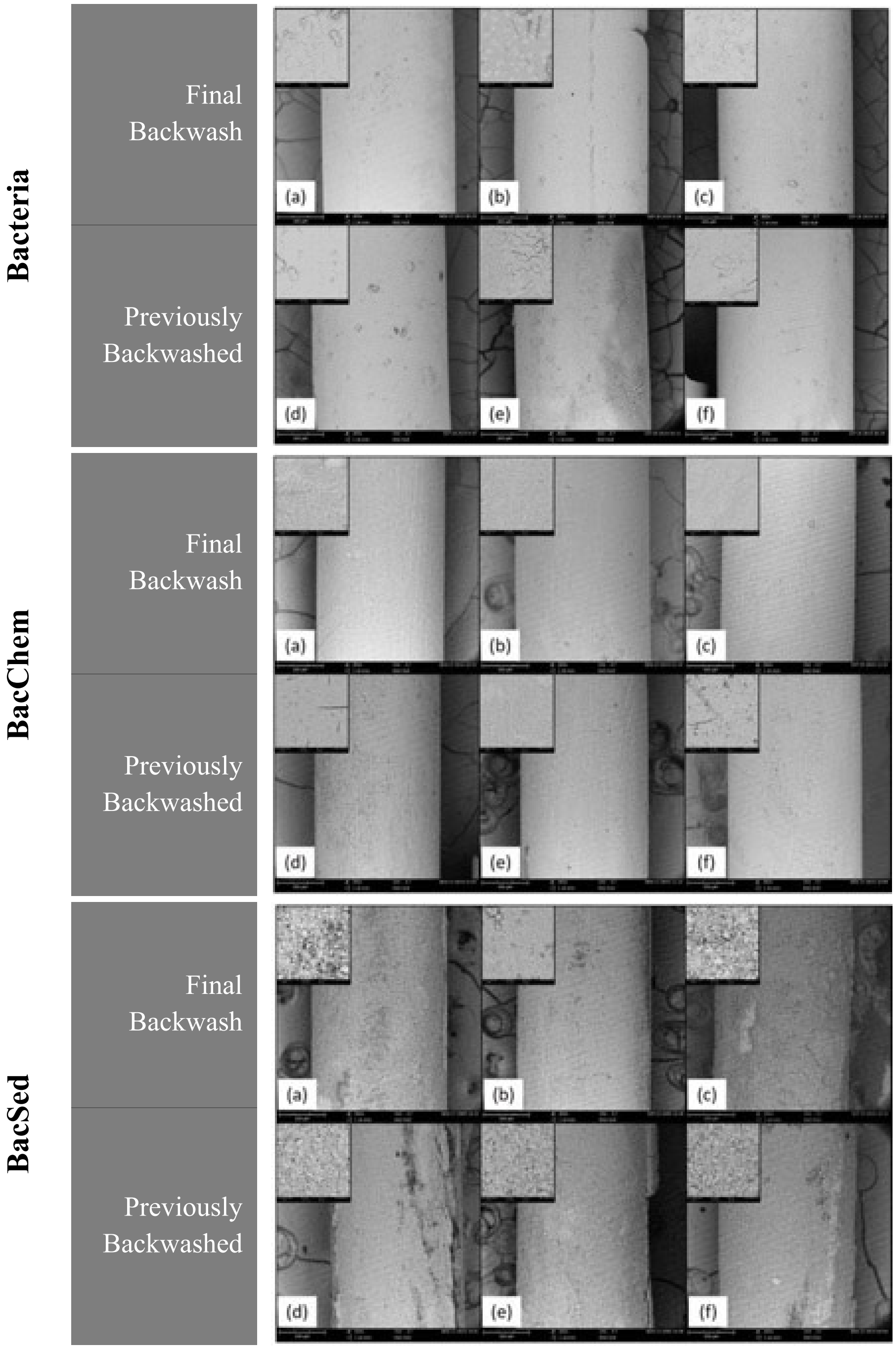


**Figure S13. SEM images of fouling control on F4 fibers by cleaning solutions**. Outer membrane surface at 500x and 15,000x (insets) showing fouling layer after filtration with final backwash with: (a) DI water, (b) bleach, (c) vinegar; without final backwash, but previously backwashed with: (d) DI water, (e) bleach, (f) vinegar.
